# Hepcidin depending on astrocytic NEO1 ameliorates blood-brain barrier dysfunction after subarachnoid hemorrhage

**DOI:** 10.1038/s41419-024-06909-x

**Published:** 2024-08-07

**Authors:** Boyang Wei, Wenchao Liu, Lei Jin, Yaxian Huang, Wenping Cheng, Haiyan Fan, Shixing Su, Fa Jin, Xin Zhang, Zeyu Yang, Shuyin Liang, Longxiang Li, Yu Wu, Yanchao Liu, Chuanzhi Duan, Xifeng Li

**Affiliations:** 1 Neurosurgery Center, Department of Cerebrovascular Surgery, The National Key Clinical Specialty, The Engineering Technology Research Center of Education Ministry of China on Diagnosis and Treatment of Cerebrovascular Disease, Guangdong Provincial Key Laboratory on Brain Function Repair and Regeneration, The Neurosurgery Institute of Guangdong Province, Zhujiang Hospital, Southern Medical University, Guangzhou, 510282 China; 2 School of Materials Science and Engineering, Key Laboratory for Polymeric Composite and Functional Materials of Ministry of Education, Sun Yat-sen University, Guangzhou, 510275 China

**Keywords:** Stroke, Blood-brain barrier, Translational research

## Abstract

Subarachnoid hemorrhage (SAH) significantly compromises the blood-brain barrier (BBB) and impairs patient recovery. This study elucidates the critical role of astrocytic Neogenin-1 (NEO1) in BBB integrity post-SAH and examines the regulatory effects of hepcidin on endothelial cell (EC) function amid NEO1-mediated disruptions in iron homeostasis. Proteomic analyses of cerebrospinal fluid (CSF) from SAH patients revealed a substantial decrease in NEO1 expression, identifying it as a key factor in BBB integrity. 111 CSF proteins were significantly reduced in early SAH stages (days 1–3), with NEO1 among the most significantly altered. This dysregulation was linked to poorer patient outcomes, as indicated by a negative correlation between NEO1 levels and Modified Rankin Scale scores six months post-SAH (*R* = −0.4743, *P* < 0.0001). Experimental models further highlighted the importance of NEO1: SAH model and NEO1^GFAP-Cre^ mice exhibited exacerbated EC dysfunction and increased BBB permeability, evidenced by significant Evans Blue retention and dextran leakage in the parietal cortex, effects that were mitigated by hepcidin administration. Our findings highlight the complex interplay between astrocytic signaling and endothelial function in SAH pathophysiology. The loss of astrocytic NEO1 led to increased EC proliferation and altered BBB structure, as confirmed by transmission electron microscopy and immunostaining for PECAM-1, indicating heightened blood vessel density in the affected cortex. Hepcidin treatment effectively reversed the EC dysfunction and BBB disruption in both NEO1-cKO mice and the SAH model, highlighting its potential as a therapeutic agent to enhance recovery and improve prognosis following SAH.

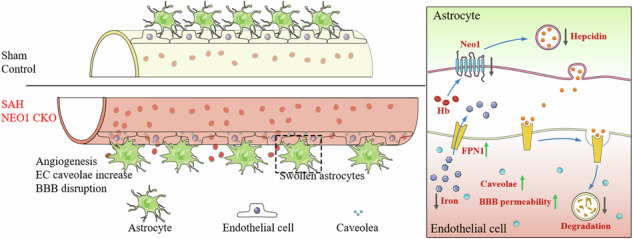

## Introduction

Subarachnoid hemorrhage (SAH) is a neurological condition, characterized by high mortality and significant morbidity due to brain aneurysm rupture. It leaves approximately 50% of survivors with considerable neurological impairments [1, 2]. Despite advancements in clinical interventions, effective management of brain edema, a primary contributor to neurological deficits and adverse prognostic outcomes, remains elusive [3]. Cerebral edema, identified as the sole prognostic factor amenable to therapeutic intervention in a comprehensive analysis of admission parameters, underscores the need for targeted research in this area [4]. Central to the pathogenesis of brain edema post-SAH is the disruption of the blood-brain barrier (BBB), a complex multicellular vascular structure essential for maintaining neuronal homeostasis [5]. Disruption of any BBB component following SAH precipitates neuroinflammation and neurodegeneration [6]. Given the propensity for SAH model mice to exhibit bleeding at the bifurcation of the external and common carotid arteries, investigations into BBB disruption have predominantly focused on the adjacent temporal base cortex [7–9]. Yet, this region’s physiological function fails to fully account for the neurological deficits observed in this model. Crucially, a study employing the apparent diffusion coefficient identified ubiquitous brain edema in SAH patients, emphasizing the need to explore BBB integrity beyond the temporal base cortex, particularly in the parietal cortex following SAH [10]. The cerebral blood vessels formed by endothelial cells (ECs) are the core element of the BBB. Research has highlighted a marked reduction in cerebral blood vessel (BV) density within the temporal base cortex at 24 h post-SAH [11, 12]. Astrocytes, with their end-feet enveloping the ECs, play an important role in maintaining BBB integrity [13]. The preservation of astrocytes has been demonstrated to forestall further BBB disruption post-SAH, although the precise mechanisms underpinning this protective effect remain to be elucidated [14]. There is accumulating evidence to suggest that astrocytes may modulate the fate of cerebral ECs via the secretion of soluble factors, yet the impact of astrocytes on endothelial cells following SAH remains uninvestigated [15–17].

By conducting proteomic analyses of cerebrospinal fluid (CSF) from SAH patients, we have identified a reduction in neogenin (NEO1), indicating its potential involvement in SAH pathophysiology. NEO1, a member of the ‘deleted in colorectal cancer’ protein family, acts as a receptor for various signaling molecules, including netrins, repulsive guidance molecules, and bone morphogenetic proteins, and is prominently expressed in both astrocytes and neurons within the brain [18]. Deficiency in NEO1 is linked to severe outcomes, such as early neonatal death and epilepsy [19, 20]. Investigations into the role of NEO1 within astrocytes, facilitated by the use of conditional knockout mice, have underscored its significance in vascular homeostasis [21]. However, no study has tested whether NEO1 participates in SAH pathophysiological mechanisms.

Galea et al. have correlated cortical iron deposits with cognitive outcomes in SAH patients [22]. Studies have shown that administering the iron chelator deferoxamine mitigates vascular dysfunction in experimental SAH models [23]. This underscores the important role of iron homeostasis in maintaining BBB integrity. Zhang et al. demonstrated that NEO1 is instrumental in regulating iron homeostasis through the upregulation of hepcidin secretion in the liver [24]. Hepcidin is a small peptide and is the main regulator of iron homeostasis. Hepcidin, a key regulator of iron metabolism, exhibits high expression in astrocytes within the brain and is known to influence iron transport across the BBB by modulating ferroportin 1 (FPN1) on brain microvascular endothelial cells (BMVECs) [25, 26]. These findings suggest a role for hepcidin in maintaining the integrity of the BBB. Nevertheless, the specific function of hepcidin in BBB maintenance post-SAH, along with the underlying mechanism of its secretion (particularly its dependence on NEO1 in the astrocytic context) remains to be elucidated.

In this study, we report a correlation between decreased levels of NEO1 in the CSF of SAH patients and poor prognostic outcomes. We delineate the mechanism through which astrocytic NEO1 deficiency exacerbates BBB disruption, utilizing NEO1 conditional knockout and SAH model mice. We further demonstrate that a reduction in NEO1 expression leads to decreased hepcidin levels and disrupted iron homeostasis in BMVECs. The administration of hepcidin was found to ameliorate BBB disruption following SAH, positioning the regulation of hepcidin by astrocytic NEO1 as a novel therapeutic strategy for protecting the BBB following SAH.

## Results

### Decreased NEO1 expression in SAH patient CSF

**Fig. 1:**
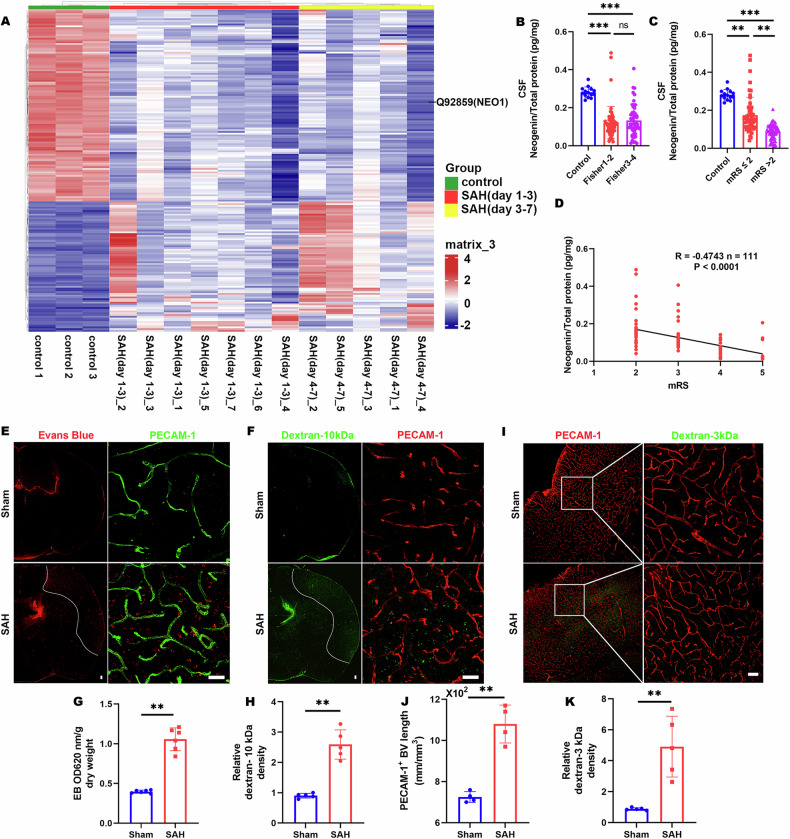
NEO1 is decreased in the CSF of SAH patients and increased blood vessel density but increased BBB leakage in the SAH mouse cortex. **A** Heatmap reveals the differentially expressed proteins from the comparisons of SAH (day 1–3) vs. control and SAH (day 4–7) vs. control, the expression of which was normalized, the high expression displayed in red and low in blue. **B** Quantification of NEO1 expression in CSF of control, SAH patients with Fisher1-2, and SAH patients with Fisher3-4. **C** Quantification of NEO1 expression in CSF of control, SAH patients with mRS ≤2, and SAH patients with mRS >2. **D** Analysis of the correlation between mRS in 6 months of SAH patients and the expression of NEO1 in CSF of SAH patients at the initial 7 days were performed with Pearson’s correlation test. **E**, **F** Representative images of EB (red) and dextran-10 kDa (green) coimmunostained with PECAM-1 antibody in sham and SAH cortex. **G**, **H** Quantitative analysis of data in (**A**) and (**B**) (*n* = 6 mice/group). **I** Representative images of dextran-3 kDa coimmunostained with PECAM-1 antibody in sham and SAH cortex. **J**, **K** Quantitative analysis of data in (**C**) (*n* = 5 mice/group). Scale bars: 20 μm. Data are presented as mean ± SD and were analyzed by student’s *t*-test, *P < 0.05; **P < 0.01; ns not significant.

To identify potential factors that may underlie BBB disruption after SAH, we conducted proteomic analyses on CSF from both healthy individuals and SAH patients. Hierarchical clustering effectively differentiated CSF samples from the various groups (control, day 1–3, and day 3–7 after SAH), demonstrating clear between-group separation (Fig. 1A). Our analysis identified 111 proteins that were significantly reduced in the SAH (day 1–3) samples compared to control samples, whereas 46 proteins showed a significant increase (Fig. S1A). Additionally, in the SAH (day 4–7) samples compared to control samples, 111 proteins were again significantly decreased, with 116 proteins exhibiting a decrease (Fig. S1B). A comparison between the early (day 1–3) and later (day 4–7) SAH stages revealed a decrease in 2 proteins (Fig. S1C). Utilizing the Dijkstra algorithm to screen for differential protein expression between SAH (days 1–3) and control samples revealed NEO1, CLSTN1, DCC, and NEGR1 as significantly altered (methods are detailed in supplementary materials). A previous study suggested the involvement of NEO1 in BBB maintenance [21]. To further elucidate the role of NEO1 in SAH, we analyzed NEO1 expression in 111 SAH patients via ELISA (Table S1), finding lower NEO1 levels in the CSF of SAH patients compared to non-SAH individuals, with no association to the modified Fisher grade (Fig. 1B), indicating that NEO1 expression in SAH patient CSF does not correlate with bleeding volume. Modified Rankin Scale (mRS) grades were obtained for these patients six months following hemorrhage, revealing significantly lower NEO1 expression in patients with mRS >2 compared to those with mRS ≤2 (Fig. 1C). A significant negative correlation was observed (*R* = −0.4743, *P* < 0.0001; Fig. 1D), linking lower NEO1 expression in CSF to poorer prognoses.

### BBB compromise and elevated PECAM-1+ blood vessel density in the parietal cortex following SAH

**Fig. 2:**
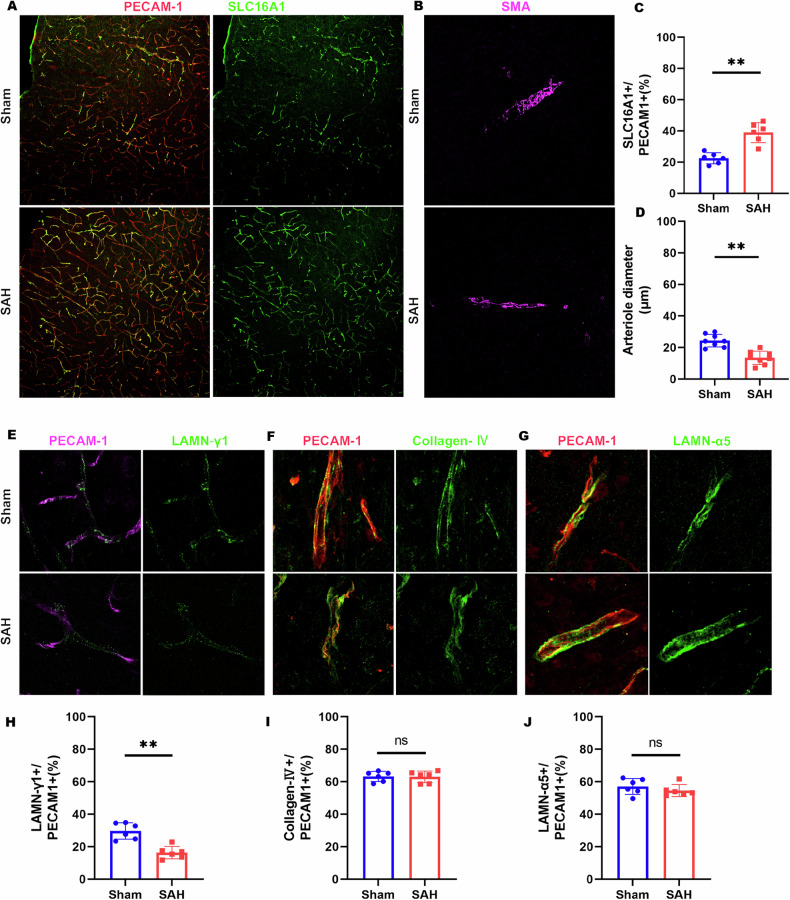
Increased vein capillaries, thinner arteries, and impaired vBMs (vascular basement membranes) in the SAH cortex. **A**, **B**, **E**–**G** Representative images of coimmunostaining analyses using corresponding antibodies in sham and SAH cortex. **C**, **D**, **H**–**J** Quantification analyses of data in SLC16A1 (**A**), SMA (**B**), LAMN-γ1 (**E**), Collagen-IV (**F**), and LAMN-α5 (**G**). Scale bars: 20 μm. Data are presented as mean ± SD (*n* = 6 mice/group). **P < 0.01; ns not significant. Student’s *t*-test.

**Fig. 3:**
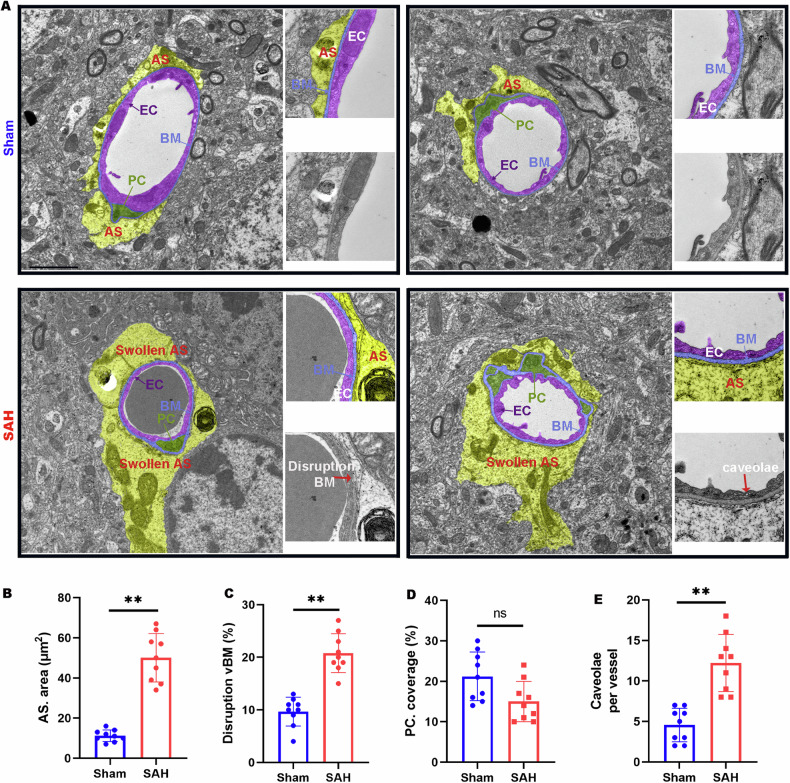
Swollen astrocytes, disrupted vBMs, and increased EC caveolae in SAH cortex by EM analysis. **A** Representative EM images of blood vessels in Sham and SAH 7 days cortex. Astrocytes are highlighted in yellow, pericytes (PC) in green, vBM in blue, and EC in purple. Scale bars: 1 μm. **B** Quantification of astrocytes area. **C** Quantification of disrupted vBMs. **D** Quantification of PC coverage. **E** Quantification of EC caveolae per vessel. Data are presented as mean ± SD (*n* = 3 vessels from 3 mice per group). **P < 0.01; ns not significant. Student’s *t*-test.

To elucidate the impact of SAH on the BBB in the parietal cortex, we utilized Evans Blue (EB) dye, injected into the tail veins thirty minutes prior to euthanasia, as a marker for assessing BBB integrity post-SAH. Control brains exhibited minimal EB accumulation, whereas significant EB retention was observed in the parietal cortex 7 days post-SAH (Fig. S2A), indicating BBB disruption. To further validate these findings, red fluorescent-labeled EB and green fluorescent-labeled dextran-10 kDa were employed, both of which showed enhanced accumulation in the SAH-affected parietal cortex (Fig. 1E–H), reinforcing evidence of BBB compromise. Given that blood vessel ECs constitute the main component of the BBB, PECAM-1 (platelet endothelial cell adhesion molecule-1), a marker for ECs, was used to assess blood vessel density in the disrupted BBB region (Fig. 1I). Interestingly, the density of PECAM-1+ vessels in the SAH model parietal cortex was found to be higher compared to the sham group, with both the length of CD31+ vessels and dextran+ signals showing a significant increase, as determined by quantitative analysis (Fig. 1J–K). To determine whether the increase in blood vessels was predominantly in the venous capillaries, we conducted immunostaining for solute carrier family 16 member 1 (SLC16A1) and SMA (smooth muscle actin). SLC16A1 is characteristically expressed in ECs of venous capillaries instead of arterioles. Following SAH, an increase in SLC16A1+ ECs was noted in the parietal cortex, while arterioles appeared thinner than in sham controls, despite arterioles not being directly associated with BBB function in the brain (Fig. 2A–D). Since vascular basement membranes (vBMs) are integral to BBB structure and synthesized by ECs, we examined key vBM components, including laminin-γ1, collagen IV, and laminin-α5. These components were uniformly distributed along the vessels in both sham and SAH groups. However, laminin-γ1 expression was notably reduced in the SAH cortex compared to the sham group (Fig. 2E–J). Transmission electron microscopy (TEM) was employed to visualize the BBB structure in the SAH-7day cortex (Fig. 3A), revealing vBM disruption, astrocyte swelling, and an increase in EC caveolae in the SAH parietal cortex (Fig. 3B–E). Further examination of EC junction proteins such as zonula occludens-1 (ZO-1), and claudin-5 via Western blot analysis showed no significant difference between the SAH and sham groups (Fig. S2B–D). These findings challenge the notion that BBB disruption in the SAH cortex results from a decrease in blood vessel ECs, suggesting instead that EC dysfunction may be the cause of BBB compromise following SAH.

### Loss of astrocytic NEO1 stimulates BV EC proliferation following SAH

**Fig. 4:**
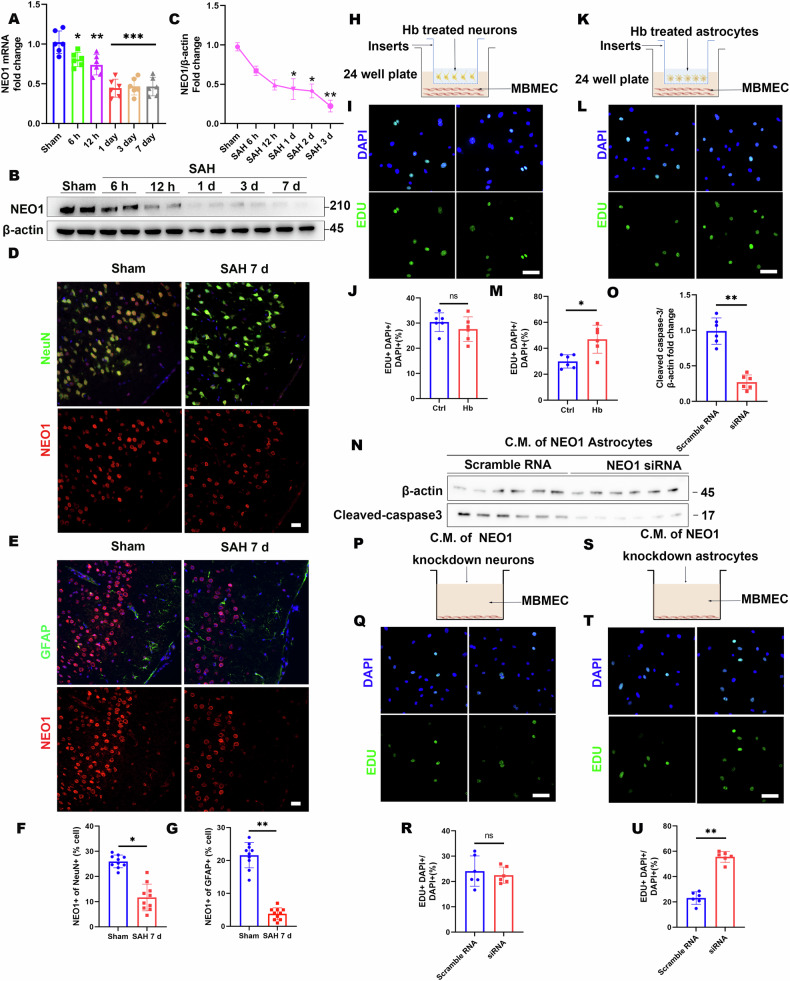
NEO1 is decreased in the SAH cortex and resulted in EC proliferation increasing and EC apoptosis reducing. **A** Total RNA of the cortex was extracted and the expression of NEO1 was quantitated by RT-qPCR (*n* = 6 mice/group). The expression level of NEO1 was normalized against β-actin. **B**, **C** Representative immunoblotting (**B**) and quantification (**C**) of NEO1 of cortex from mice subjected to SAH for different time points and sham surgery (*n* = 6 mice/group). β-actin was used as a loading control. **D**, **E** Representative images of coimmunostained NEO1 and Neun (**D**) or GFAP (**E**) in sham and SAH 7 days cortex (*n* = 10 mice/group). **F**, **G** Quantification of NEO1 in neuron (**F**) and astrocytes (**G**). **H**–**J** MBMEC cocultured with Hb-treated neurons (*n* = 6 experiments with Hb-treated neurons and its control group neurons). **K**–**M** Reduced proliferation in MBMEC cocultured with Hb-treated astrocytes (*n* = 6 experiments with Hb-treated astrocytes and its control group astrocytes). **N** Representative immunoblotting of cleaved-caspase3 from MBMEC cocultured with CM of NEO1 knockdown astrocyte. **O** Quantitative analysis of data in (**G**). β-actin was used as a loading control (*n* = 6 experiments with NEO1 knockdown astrocytes and its control group astrocytes). **P**–**R** MBMEC cocultured with NEO1 knockdown neurons (*n* = 6 experiments with NEO1 knockdown neurons and its control group neurons). **S**–**U** MBMEC cocultured with NEO1 knockdown astrocytes (*n* = 6 experiments with NEO1 knockdown astrocytes and its control group astrocytes). Scale bars: 20 μm. Data are presented as mean ± SD. *P < 0.05; **P < 0.01, ***P < 0.01. 1-way ANOVA with Tukey’s correction for multiple comparisons (**A**, **C**). Student’s *t*-test.

We induced SAH in mice to assess the transcriptional levels of NEO1 in the parietal cortex at various time points post-SAH via RNA sequencing. The findings revealed a consistent decrease in NEO1 mRNA expression at 1 day, 3 days, and 7 days following SAH (Fig. 4A). Western blot analysis corroborated a time-dependent decline in NEO1 expression from day 1 to day 7 following SAH (Fig. 4B, C). Immunostaining was employed to localize NEO1 expression across different brain cell types, demonstrating its presence in both astrocytes and neurons on the cortical surface. Notably, NEO1 expression diminished in GFAP+ astrocytes and NEUN+ neurons at SAH-7 days (Fig. 4F, G). To determine the influence of astrocytic versus neuronal NEO1 on blood vessel ECs, coculture assays were conducted. Mouse brain microvascular ECs (MBMECs) were cocultured with either primary astrocytes or neurons treated with hemoglobin (Hb) in a Transwell assay. Identification pictures of primary astrocytes and primary neurons are shown in Fig. S3A. Following Hb treatment, a reduction in NEO1 expression was observed in both primary astrocytes and neurons (Fig. S3B–D). Incorporation of 5-ethynyl-2′-deoxyuridine (EdU) into the medium 4 h before culture termination revealed an increased number of EdU+ ECs when cocultured with Hb-treated astrocytes, as opposed to those cocultured with control astrocytes (Fig. 4I, J). However, no significant change was detected in the number of EdU+ ECs when cocultured with Hb-treated neurons compared to control neurons (Fig. 4L, M). To further determine the impact of astrocytic NEO1 on cortical EC proliferation, we cocultured ECs with astrocytes and neurons subjected to NEO1 knockdown via siRNA (efficacy verified through PCR and Western blot; Fig. S4A–C). EdU staining of ECs cocultured with NEO1 knockdown astrocytes mirrored the proliferation observed with Hb-treated astrocytes (Fig. S3E–I), suggesting that EC proliferation is enhanced in the absence of astrocytic NEO1. Given the lack of direct contact between ECs and astrocytes in the Transwell coculture assay, this proliferative effect likely stems from the effects of secreted factors from NEO1 knockdown astrocytes. Subsequent detection of cleaved-caspase3 in cultured MBMECs with conditioned medium (C.M.) from NEO1 knockdown astrocytes was minimal (Fig. 4N, O). To validate this hypothesis, MBMECs were cultured with C.M. from control and NEO1 knockdown astrocytes and neurons (Fig. 4P, S). EC proliferation was significantly elevated in the presence of C.M. from NEO1 knockdown astrocytes (Fig. 4Q, T, R, U), supporting the notion that the loss of astrocytic NEO1 contributes to EC proliferation via secreted factors, demonstrating a novel mechanism through which astrocytic signaling influences vascular dynamics following SAH.

### BBB disruption in astrocytic NEO1 conditional knockout mice

**Fig. 5:**
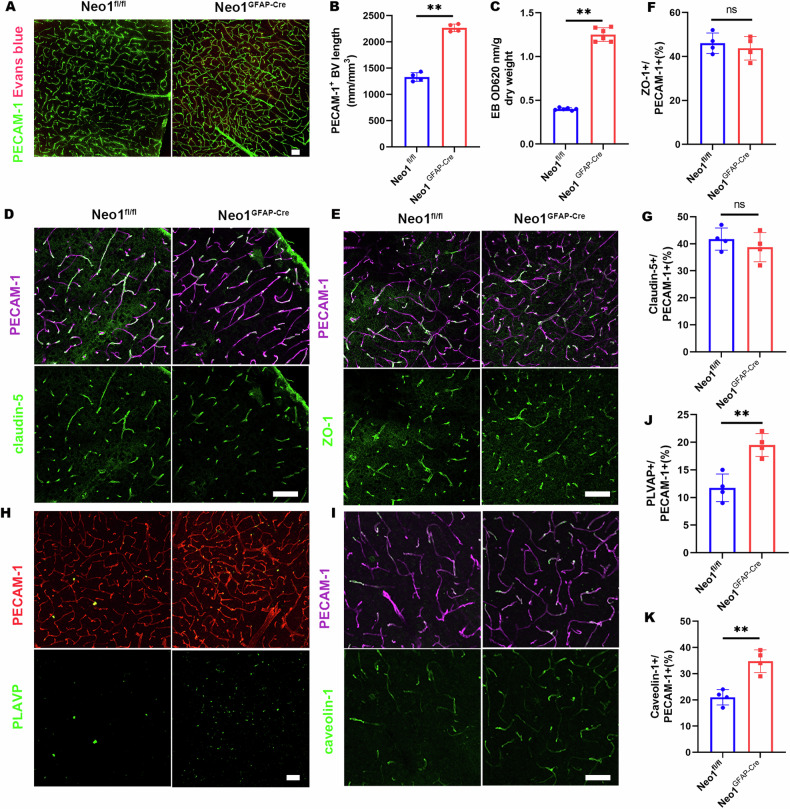
Impaired EC barrier, increased blood vessel density, and increased BBB leakage in NEO1^GFAP-Cre^ cortex. **A** Representative images of EB (red) coimmunostained with PECAM-1 antibody in NEO1^fl/fl^ and NEO1^GFAP-Cre^ cortex (Imaged at P30). **B**, **C** Quantitative analyses of BV length (**B**) and EB extravasation (**C**). **D**, **E** Representative images of claudin-5 (**D**) and ZO-1 (**E**) coimmunostained with PECAM-1 antibody in NEO1^fl/fl^ and NEO1^GFAP-Cre^ cortex. **F**, **G** Quantitative analyses of data in (**D**) and (**E**). **H**, **I** Representative images of PLVAP (**H**) and caveolin-1 (**I**) coimmunostained with PECAM-1 antibody in NEO1^fl/fl^ and NEO1^GFAP-Cre^ cortex. PLVAP is a maker for EC fenestrae, caveolin-1 is an EC caveolae marker. **J**, **K** Quantitative analyses of data in (**H**) and (**I**). Scale bars: 20 μm. **P < 0.01; ns not significant. Student’s *t*-test.

**Fig. 6:**
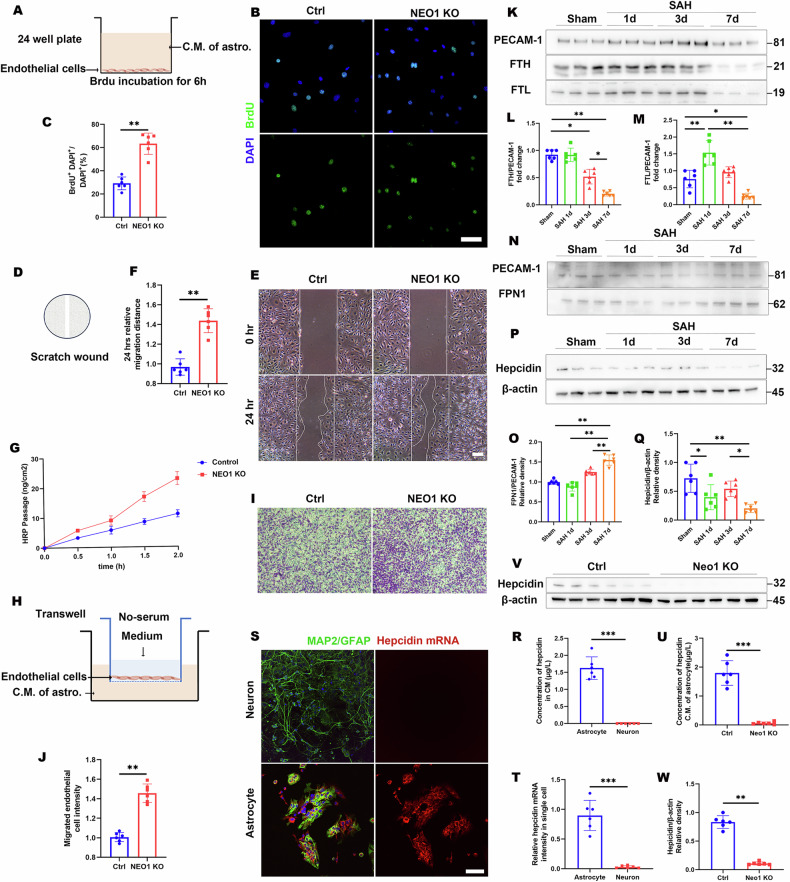
Increased EC migration in HUVEC cultures exposed to CM of NEO1 KO astrocytes and hepcidin expression needs NEO1. **A**–**C** Increased EC proliferation in HUVEC cocultured with CM of NEO1-KO astrocytes. EDU incubated for 4 h (*n* = 6 experiments of astrocytes from NEO1 GFAP-Cre and its control group). **D**–**F** Increased EC migration in HUVEC cocultured with CM of NEO1-KO astrocytes (*n* = 6 experiments of astrocytes from NEO1^GFAP-Cre^ and its control group). **G** The permeability of HUVEC monolayers (*n* = 3 experiments of astrocytes from NEO1^GFAP-Cre^ and its control group). **H**–**J** Transwell assay to access HUVECs migration. **K**–**Q** Representative immunoblotting (**K**–**M**) and quantification (**L**, **M**, **O**, and **Q**) of FTH, FTL, FPN1, and hepcidin of cortex endothelial cells from mice subjected to SAH for different time points and sham surgery (*n* = 6 mice/group). PECAM-1 was used as a loading control. **R** The Quantification analysis of hepcidin in CM of primary astrocytes and primary neurons by ELISA assay (*n* = 6 experiments/group). **S**, **T** FISH analysis of hepcidin mRNA in primary astrocytes and primary neurons (*n* = 6 experiments/group). Scale bars: 20 μm. **U** Quantification analysis of hepcidin in CM of astrocytes from NEO1^GFAP-Cre^ and its control group by ELISA assay (*n* = 6 experiments/group). **V**, **W** Representative immunoblotting (**V**) and quantification (**W**) of hepcidin in astrocytes from NEO1^GFAP-Cre^ and its control group (*n* = 6 experiments of astrocytes from NEO1^GFAP-Cre^ and its control group). Data are presented as mean ± SD. *P < 0.05; **P < 0.01, ***P < 0.01. 1-way ANOVA with Tukey’s correction for multiple comparisons (**B**, **C**, **E,** and **G**). Student’s *t*-test.

Astrocytic NEO1 conditional KO (cKO) mice were generated by crossing NEO1^fl/fl^ with GFAP-cre mice leading to the selective ablation of NEO1 in astrocytes. These NEO1-cKO mice exhibited BBB deficits and an increase in parietal cortical blood vessels, mirroring the changes observed in SAH model mice 7 days post-hemorrhage (Figs. 5A–C, and S5A–C). To delve deeper into the role of astrocytic NEO1 in BBB integrity, we employed transmission electron microscopy (TEM) for ultrastructural analysis (Fig. S6A). Notably, astrocytes in the NEO1-cKO cortex appeared swollen (Fig. S6B), while vascular basement membranes (vBMs) and pericyte coverage remained unchanged compared to controls (Fig. S6C, D). An increase in caveolae vesicles was observed in the NEO1-cKO cortex (Fig. S6E), highlighting altered endothelial cell (EC) morphology. However, endothelial cell tight junctions also are attributed to BBB maintenance, which cannot be detected by TEM. Despite these changes, the integrity of endothelial cell tight junctions, essential components of BBB maintenance, appeared unaffected in NEO1-cKO mice, as indicated by the unaltered expression of tight junction markers ZO-1 and claudin-5, both in immunohistochemical analysis and western blotting (Figs. 5D–G, S5D–F). This suggests that tight junctions may not be the primary factor in BBB disruption within this model. Further examinations were then directed to the caveolae and fenestrae, structures critical to EC barrier function. Markedly, levels of caveolin-1 (a caveolae marker) and plasmalemma vesicle-associated protein (PLVAP, an EC fenestrae marker) were elevated in NEO1-cKO mice compared to controls (Fig. 5H–K), indicating changes in EC functional properties. To assess the impact of astrocytic NEO1 deficiency on EC function, coculture assays with human umbilical vein endothelial cells (HUVEC) and conditioned medium (CM) from control and NEO1 KO astrocytes were performed (Fig. 6A). These assays revealed that HUVEC proliferation increased when cocultured with CM from NEO1-KO astrocytes (Fig. 6B, C). Moreover, wound healing and Transwell migration assays demonstrated enhanced HUVEC migration in the presence of CM from NEO1 KO astrocytes (Fig. 6D–F), suggesting augmented angiogenic activity. Furthermore, horseradish peroxidase (HRP), used as a marker molecule to assess macromolecular permeability of HUVEC monolayers, showed increased permeability in cells cocultured with CM from NEO1 KO astrocytes as early as 0.5 h (Fig. 6G). These findings highlight the critical role of astrocytic NEO1 in maintaining cortical blood vessel integrity and suggest that deficiency of astrocytic NEO1 may lead to BBB disruption by altering astrocytic secreted factors, thereby affecting EC function and contributing to vascular anomalies.

### BBB disruption linked to ECs iron homeostasis in SAH model and NEO1-cKO mice

The observed EC barrier deficits in NEO1-cKO mice are consistent with prior findings [21], which suggested that inducing astrocytic Netrin-1 (NTN1) expression could mitigate BBB disruption in the NEO1-cKO mice cortex. Investigating this potential for BBB repair post-SAH, we administered AAV-GFAP-NTN1 into the parietal cortex of mice prior to SAH induction and monitored them for 14 days (Fig. S7A). Despite GFP fluorescence indicating successful injection, PECAM-1 immunostaining revealed no significant differences in blood vessel (BV) density or dextran-10 kDa leakage between the control virus SAH-7 days model and the AAV-GFAP-NTN1 SAH-7 days model (Figs. S7B and S8A), indicating that increasing astrocytic NTN1 does not rectify the SAH-induced cortical blood vessels deficits.

Given prior demonstrations that modulating perivascular iron levels improves outcomes post-SAH by influencing blood vessels [23], we explored the relevance of increased blood vessel density in the SAH model parietal cortex to iron homeostasis. Ferritin-H (FTH) and Ferritin-L (FTL), iron storage proteins, were analyzed in isolated ECs from the mice parietal cortex via Western blot (Fig. 6K), showing a slight increase 1 days post-SAH but a significant decrease 7 days post-SAH (Fig. 6L, M). With FPN1 playing a critical role in the release of iron from ECs, we assessed FPN1 expression in correlation with cortical EC iron storage protein levels post-SAH (Fig. 6N, O).

Hepcidin, crucial for brain iron homeostasis through its interaction with FPN1 [27, 28], was examined to determine its association with FTH and FTL levels in ECs post-SAH. Western blot analysis revealed a decrease in hepcidin expression at 3 and 7 days post-SAH, concurrent with changes in FTH and FTL (Fig. 6P, Q). In vitro, hepcidin levels significantly declined in response to Hb treatment in primary astrocytes (Fig. S8E, F), yet the primary source of hepcidin in the brain remained uncertain. To identify the cellular source of hepcidin within the brain, we initially assessed hepcidin levels in the CM of cultured primary astrocytes and neurons. Hepcidin was nearly undetectable in the CM derived from primary neurons (Fig. 6R). To further substantiate this finding, we utilized fluorescence in situ hybridization (FISH) to analyze the expression of *Hamp*, the gene encoding hepcidin, in both primary astrocytes and neurons. The analysis revealed *Hamp*-positive signals exclusively within primary astrocytes, with no such signals detected in neurons (Fig. 6S, T), indicating a predominant astrocytic contribution to hepcidin production. Considering previous research indicating the necessity of Neogenin-1 (NEO1) for hepcidin expression in hepatocytes [29], the role of NEO1 in astrocytic hepcidin expression within the brain required clarification. To this end, primary astrocytes derived from NEO1-cKO mice and their control counterparts were cultured. ELISA analysis of the CM from NEO1-cKO mouse-derived astrocytes revealed an absence of hepcidin expression (Fig. 6U), in stark contrast to control astrocytes. Subsequent Western blot analysis confirmed the presence of hepcidin in the CM from control astrocytes, but not from those derived from NEO1-cKO mice (Fig. 6V, W). These results demonstrate the critical role of NEO1 in astrocytes for hepcidin production.

### Hepcidin mitigates BBB disruption in NEO1-cKO mice and the SAH model

**Fig. 7:**
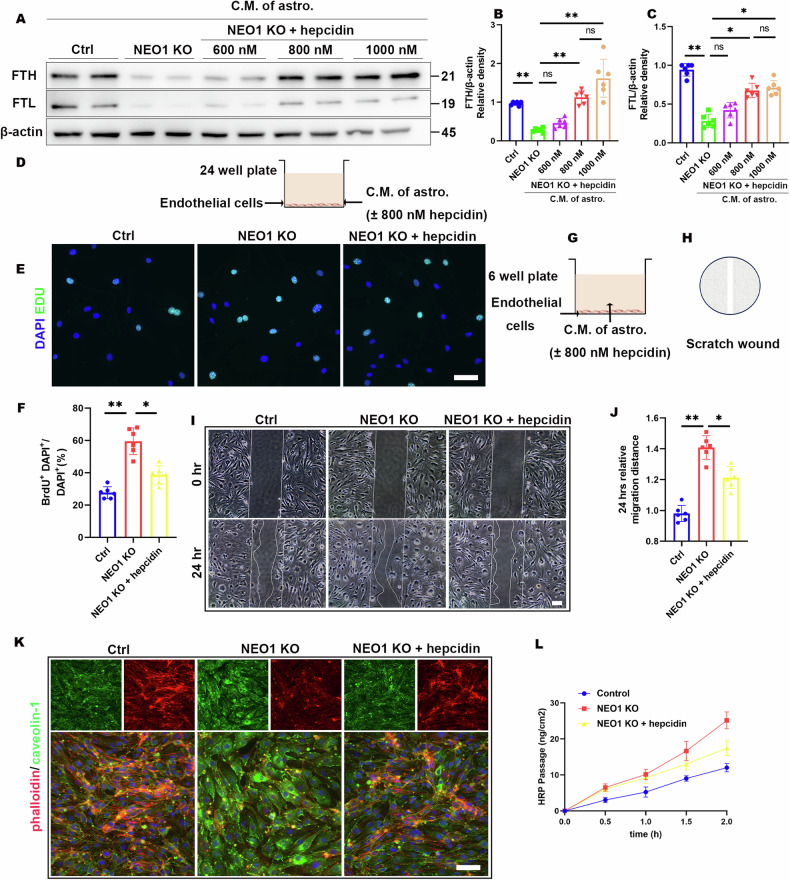
Hepcidin alleviation of EC iron storage decreases, proliferation, migration, and leakage induced by CM of astrocytes from NEO1^GFAP-Cre^. **A**–**C** Representative immunoblotting (**A**) and quantification (**B**, **C**) of hepcidin effect on FTH and FTL of MBMEC in the presence of CM of astrocytes. Data are presented as mean ± SD (*n* = 6 experiments/group). **D**–**F** Hepcidin (800 nM) inhibition of MBMEC proliferation. **D** Schematic of hepcidin administration in MBMEC cultures in the presence of CM of NEO1 KO astrocytes. EDU incubated for 4 h. **E** Representative images of EDU + MBMEC. **F** Quantitative analysis of data in (**E**). Data are presented as mean ± SD (*n* = 6 experiments/group). **G**–**I** Hepcidin inhibition of MBMEC migration. **G**, **H** Schematic of hepcidin administration in MBMEC cultures in the presence of CM of NEO1 KO astrocytes to access migration by wound-healing assay. **I** Representative images of MBMEC migration 24 h after wound scratching. **J** Quantification analysis of data in (**H**). Data are presented as mean ± SD (*n* = 6 experiments/group). **K** Representative images of caveolae of MBMEC. **L** Hepcidin inhibition of permeability of MBMEC monolayers. Data are presented as mean ± SD (*n* = 3 experiments/group). *P < 0.05; **P < 0.01; ns not significant. 1-way ANOVA with Tukey’s correction.

To explore the effects of hepcidin on ECs, we first determined the concentration of hepcidin by the ECs and CM of NEO1 KO astrocyte coculture Transwell assay. in a coculture Transwell assay involving ECs and conditioned medium (CM) from NEO1 knockout (KO) astrocytes. Guided by prior research of hepcidin effects in *vitro*, concentrations of 600 nM, 800 nM, and 1000 nM hepcidin were introduced into the coculture medium [30]. We observed that hepcidin administration led to an increase in EC iron content, with no significant difference in iron storage proteins (FTH and FTL) between the 800 nM and 1000 nM groups (Fig. 7B, C). Furthermore, the influence of 800 nM hepcidin on EC proliferation was investigated, revealing a reduction in EdU+ MBMECs when cocultured with CM from NEO1 KO astrocytes (Fig. 7D–F). Hepcidin also reduced EC migration in response to CM from NEO1 KO astrocytes (Fig. 7G–J) and significantly mitigated the increase in caveolae in MBMECs induced by CM from NEO1 KO astrocytes, as shown by IF analyses (Fig. 7K). Importantly, hepcidin improved the permeability of the MBMEC monolayer in coculture with CM from NEO1 KO astrocytes (Fig. 7L).

To determine whether hepcidin could rectify the BBB disruption in NEO1-cKO mice, hepcidin was injected into the left cortex of NEO1-cKO mice, with PBS administered to the contralateral side as a control (Fig. S9A). In the hepcidin-treated parietal cortex, BV density and dextran 3 kDa leakage were significantly reduced compared to the control side, as evidenced by immunostaining (Fig. S9B, C). Additionally, cortical EC caveolae were reduced in the hepcidin-injected cortex (Fig. S9D–F), and hepcidin reversed the CM-induced decrease in MBMEC iron content from NEO1 KO astrocytes (Fig. S9G–I), showcasing hepcidin’s effectiveness in reducing abnormal BV angiogenesis and reducing BBB disruption in the NEO1-cKO cortex.

**Fig. 8:**
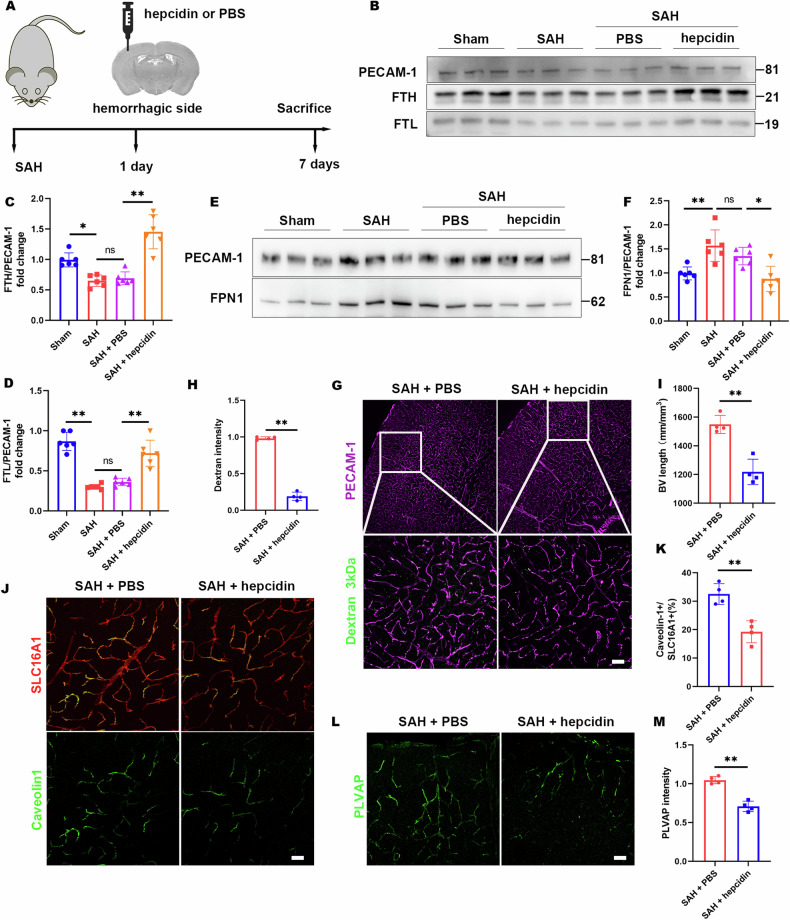
Hepcidin amelioration of blood vessel increase and BBB leakage in the cortex of the SAH model. **A** Schematic of hepcidin administration in SAH cortex. Hepcidin was injected into the hemorrhagic side cortex of the SAH mice model. **B** Representative immunoblotting of hepcidin effect on FPN1 of EC in SAH cortex. **C** Representative immunoblotting of hepcidin effect on FTH and FTL of EC in SAH cortex. **D**–**F** Quantitative analysis of data in (**A**) and (**B**) (*n* = 6 mice/group). 1-way ANOVA with Tukey’s correction for multiple comparisons. **G** Representative images of dextran 3 kDa leakage in the hepcidin-injected and PBS-injected cortices. **H**, **I** Quantitative analyses of dextran leakage and BV length. **J** Representative images of caveolin-1 coimmunostained with SLC16A1. **K** Quantitative analysis of data in (**J**). **L** Representative images of PLVAP staining in the cortex. **M** Quantitative analysis of data in (**E**). Data are presented as mean ± SD (*n* = 4 mice/group). Scale bars: 20 μm. *P < 0.05; **P < 0.01. Student’s *t*-test.

To test the effect of hepcidin on cortical BVs after SAH, hepcidin was administered into the parietal cortex on the hemorrhagic side one-day following SAH. Examination at seven days post-SAH revealed that hepcidin treatment increased FTH and FTL levels in cortical ECs compared with controls (Fig. 8B–D), with corresponding adjustments observed in FPN expression (Fig. 8E, F). The dextran 3 kDa tracer assay further substantiated the positive impact of hepcidin on BBB integrity, showing decreased BV density and dextran leakage in the hepcidin-treated cortex relative to the control (Fig. 8G–I). Additionally, fenestrae and caveolae in cortical ECs were notably reduced following hepcidin treatment (Fig. 8J–M). Taken together, these results suggest that hepcidin can ameliorate the parietal cortex BBB disruption after SAH (as shown in the schematic diagram of Fig. 9).

## Discussion

BBB disruption is a critical determinant of secondary injury after SAH, with endothelial cells, the most important element of the BBB, being particularly vulnerable post-SAH. Earlier research pinpointed MMP9, secreted by astrocytes, as a disruptor of vBMs which are integral to BBB integrity [9]. Despite the recognized astrocyte support for endothelial cells, the intricate mechanisms mediating their interaction post-SAH have remained elusive. Our study sheds light on the role of astrocytic NEO1 in modulating endothelial cell function after SAH, demonstrating that NEO1-driven hepcidin secretion from astrocytes critically influences endothelial iron homeostasis and functionality.

BBB disruption is a hallmark of SAH, with most investigations focusing on the temporal base cortex due to common hemorrhage sites in SAH models [31, 32]. However, clinical observations indicate widespread brain edema regardless of the initial bleed location, suggesting that disruptions confined to the temporal base cortex cannot fully account for the observed motor and sensory deficits in SAH model mice. We documented BBB disruption in the parietal cortex 7 days post-SAH, attributing brain injury primarily to hemosiderin accumulation from blood following the SAH [33]. Previous research traced blood migration from the temporal base to the sensory cortex via perivascular spaces in SAH models [34], implicating free iron in vascular damage post-SAH [23]. Initially, vBM disruption and blood vessel reduction were considered primary causes of early BBB disruption post-SAH [35, 36], yet the underlying mechanisms beyond 3 days post-SAH remained unexplored. Our analysis of vBM key components (LAMN-γ1, LAMN-α5, and collagen IV) revealed a specific decline in LAMN-γ1 in the sensory cortex 7 days post-SAH, while endothelial cell tight junction proteins (claudin-5, ZO-1) did not exhibit significant changes. This suggests that endothelial cell proliferation, potentially as a reparative response to initial SAH-mediated damage, contributes to later-stage BBB disruption, alongside increased fenestration and caveolae in cortical ECs, highlighting impaired EC barrier function as a central cause of BBB disruption in the parietal cortex.

We observed that NEO1, belonging to the DCC family of netrin membrane receptors, influenced astrocyte swelling, EC proliferation, caveolae formation, and BBB disruption in NEO1 GFAP-Cre mice, mirroring the 7-day post-SAH phenotype. have documented that AAV-GFAP-NTN1 administration could alleviate BBB disruption in Neo KO cortex [21], the role of NTN1, a key protein in regulating neural stem cell migration within the adult CNS through its interaction with the NEO1 receptor [37], merits further examination. Research by Kun Xiong highlighted that treatment with recombinant NTN1 protein could reduce neuron ferroptosis following SAH, yet it did not demonstrate reparative effects on BBB disruption [38]. In our study, despite augmenting NTN1 expression in astrocytes, the injection of AAV-GFAP-NTN1 failed to rectify the permeability deficits observed in the BBB of mice 7 days post-SAH. Previous findings have indicated that NTN1 knockout in astrocytic mice leads to a reduction in endothelial tight junction proteins, precipitating BBB disruption [39]. Nonetheless, these phenomena were not observed in the parietal cortex 7 days post-SAH in our investigation, suggesting that NTN1 may not play a critical role in BBB disruption at this stage following SAH. This insight challenges the presumed importance of NTN1 in the context of acute phase BBB disruption post-SAH, necessitating a reevaluation of its mechanistic involvement and therapeutic potential in BBB integrity.

Hepcidin, an iron-regulatory hormone, is instrumental in managing iron transport across the BBB [40], only interacting with its endothelial cells [41]. Zechel et al. first demonstrated the expression widely of hepcidin mRNA in many brain regions [42], noting its presence in both astrocytes and neurons. Despite ongoing debates regarding the precise origin of hepcidin within the brain, especially in the context of neurodegenerative disorders [43], our findings pinpoint astrocytes, not neurons, as the primary site of hepcidin mRNA expression. Additionally, hepcidin protein was not detectable in Neo1 KO astrocytes, aligning with literature suggesting that the cytoplasmic domain of Neo1 is crucial for hepcidin expression in hepatocytes [29]. This does not contradict findings by another study, which linked iron accumulation in the microglia and neurons of SAH patients’ brain gray matter, observed 6 months after the event, with cognitive outcomes [22], as our study focuses on iron dynamics within endothelial cells. Endothelial cells, pivotal for iron transport into brain tissue, require FPN1 on their membranes to facilitate this process [44]. FPN1 represents the sole channel for iron efflux from endothelial cells [45]. Our observations revealed a precipitous decline in hepcidin, the singular protein capable of degrading FPN1, following SAH, alongside a concurrent upregulation of FPN1 expression in endothelial cells. This aligns with findings from Yan-zhong Chang, who demonstrated that astrocyte-specific hepcidin knockdown elevated brain iron levels, while FPN1 knockout in BMVECs diminished iron content in the cortex [46], suggesting potential long-term iron accumulation in the brain parenchyma post-SAH. Yet, investigations specifically focusing on endothelial cell iron content following stroke are lacking.

Despite a reduction in hepcidin expression from day 1 post-SAH, the iron content within endothelial cells slightly increased from day 1 to day 3 after SAH, a finding that is consistent with previously published work [47]. Iron, released into the subarachnoid space following SAH, undergoes degradation within the first three days. This process results in an elevation of iron content within endothelial cells, despite an increase in iron exportation from these cells during the same timeframe. Excessive iron is known to contribute to the production of reactive oxygen species, precipitating cellular death [48]. Numerous studies highlight the critical role of reducing endothelial cell iron content to mitigate cell death in various diseases [49–51]. The vital role of iron in cellular health is evident; iron deficiency within endothelial cells stimulates proliferation and anti-apoptotic mechanisms. Our observations reveal that endothelial cells are functionally disordered when their iron content diminishes due to hepcidin depletion.

Distinct from peripheral endothelial cells, brain endothelial cells exhibit a significantly reduced rate of transcytosis, essential for the selective permeability of the BBB [52]. Caveolae are particularly expressed in endothelial cells. The size of caveolar vesicles and the specific receptors within caveolae controlled the selective permeability of endothelial cells [53]. Caveolae appear to form larger and vesicular structures as part of the angiogenic response [54]. Caveolae, crucial structures within brain endothelial cells for facilitating transcytosis, are integral to maintaining BBB integrity [55]. In our study, an increase in caveolae within cortical endothelial cells was noted in both SAH model mice and Neo1-cKO mice, identifying this as the primary cause of BBB disruption in the cortical region. Evidence suggests hepcidin is a key regulator of caveolae formation in brain endothelial cells, with its deletion resulting in iron deficiency and subsequent activation of the transferrin receptor system. This activation promotes caveolae formation, enhancing transcytosis [56–58]. Our preclinical experiments with SAH model mice have confirmed that administering hepcidin reduces caveolae formation in endothelial cells, thereby alleviating BBB permeability. However, the specific mechanisms by which hepcidin influences caveolae formation in brain endothelial cells require further investigation. Furthermore, natural hepcidin is exorbitantly expensive for human application, because of the high synthesis costs. These issues have to be addressed before hepcidin can be directly applied to the clinical.

In this study, we identify cerebrospinal fluid Neo1 as a biomarker for poor prognosis in SAH patients, linking BBB disruption to endothelial dysfunction mediated by astrocytic Neo1. We elucidate how astrocytic Neo1 impacts endothelial cell iron storage and function via hepcidin, underscoring hepcidin’s protective role in maintaining BBB integrity. This highlights the potential of targeting hepcidin as a therapeutic strategy for BBB repair following SAH.

## Materials and methods

### Human cerebrospinal fluid sample collection

We utilized a cohort of 111 patients with SAH (subarachnoid hemorrhage) in this study recruited from Zhujiang Hospital, the Second Affiliated Hospital of Southern Medical University, from October 2017 to July 2021 (approval number 2023-KY-188-01). Informed consent was obtained from all patients. This work has been carried out under The Code of Ethics of the World Medical Association (Declaration of Helsinki). We excluded patients with a history of central nervous system diseases, those with serious comorbidities prior to the SAH event, or individuals who experienced other organ dysfunctions within the six months leading up to their inclusion in the study. CSF was obtained from lumbar puncture for SAH. Control CSF samples were collected from patients undergoing intraspinal anesthesia for surgeries unrelated to neurological diseases.

Upon collection, CSF samples were immediately processed by centrifugation at 3000 RPM for 15 min at a temperature of 4 °C to eliminate cells, and subsequently stored at −80 °C. Comprehensive data on patient baseline characteristics, clinical presentations, and outcomes were systematically collected and integrated from patients’ case histories.

### Animal

Both male and female C57Bl/6 mice were acquired from the Guangdong Province Animal Center. WT mice were used to make the SAH model. Neo1 flox/flox (C57Bl/6) and Neo1 GFAP-Cre (C57Bl/6) mice were supplied by Cyagen Biosciences (Guangzhou, China). The Ethics Committee of Zhujiang Hospital of Southern Medical University approved all experimental protocols (approval number LAEC-2022-009). The mice were housed at the Zhujiang Hospital Animal Experiment Center of Southern Medical University with controlled temperature and humidity conditions.

### SAH mouse model

The SAH mouse model was established using the endovascular puncture method, as previously detailed [59]. Mice weighing 25–30 g were selected for the procedure. Under anesthesia induced by 1.0–1.5% isoflurane and supplemented with O_2_, a sharpened nylon suture was introduced to perforate the internal carotid artery (ICA) via the left external carotid artery. For sham-operated mice, sutures were inserted into the ICA without puncturing any blood vessels. Vital parameters, including mean arterial pressure, partial pressure of oxygen, and heart rate, were meticulously monitored during the surgery. Procedures on all SAH model mice were executed by an investigator experienced in the technique and blinded to the SAH subgroups. Both male and female mice were incorporated into the study. To ensure consistency in the severity of SAH, a grading system was employed, dividing the basal cistern into six segments as outlined in prior research [60]. The total SAH grade, the sum of scores from all six segments, was calculated, with mice exhibiting an SAH grade lower than 8 being excluded from further analysis.

### Primary neuron culture and treatment

Primary mouse neuron cultures were established following protocols previously outlined [61]. Briefly, a pregnant mouse at E15.5 was euthanized with CO_2_. Subsequently, the fetuses were extracted and decapitated, with the heads placed in cold DMEM. Using microforceps, the meninges were carefully dissected away. The brain cortex was then excised and transferred into an Eppendorf tube containing 1.0 mL of cold DM. The cells dissociated from the cortex were cultured in neurobasal medium (Thermo Fisher Scientific Inc, Waltham, MA, USA) supplemented with 2% B-27 and 0.5 mM glutamine. This culture was maintained in a humidified incubator set to 5% CO2 at 37 °C. Primary neurons were identified using anti-MAP2 immunofluorescence. To simulate hemorrhagic conditions in vitro, heme (100 μM, Sigma) was introduced to the cultured neurons for a duration of 12 h.

### Primary astrocyte culture and treatment

Primary astrocytes were cultured following established protocols [62]. These cells were isolated from the cortex of newborn mice, aged less than 24 h. The cortical tissue, after removal of brain membranes, was subjected to digestion using 2.5% trypsin (Gibco, Grand Island, NY, USA) to dissociate the tissue into individual cells. The digestion process was halted using a complete medium, composed of DMEM supplemented with penicillin-streptomycin solution (Gibco) and 10% fetal bovine serum (FBS) (Gibco). Following centrifugation (300 × *g* for 5 min) to pellet the cells, the cell precipitate was resuspended in the complete medium and then placed in an incubator set at 37 °C with 5% CO2. Primary astrocytes were identified using anti-GFAP immunofluorescence. To simulate hemorrhagic conditions in vitro, heme (100 μM, Sigma) was introduced to the cultured astrocytes for a duration of 12 h.

### Tracer (EB and dextran) injections

To evaluate blood-brain barrier (BBB) permeability, Evans Blue (EB) dye (MilliporeSigma) and FITC-dextran molecules of 10 kDa and 70 kDa sizes (Thermo Fisher, D3305, D1821, D1823) were administered intravenously through the tail veins of live mice. The administered dose for each tracer was set at 0.2 mg/kg. Following the injection, mice were anesthetized using anisoflurane overdose and euthanized in accordance with previously established protocols [63]. After perfusion of the PBS, brain specimens were then fixed in 4% paraformaldehyde (PFA) overnight and subsequently sectioned into sections of 70 μm thickness utilizing the Leica Vibratome System. For immunostaining analysis, six sections from each brain, spanning from the forebrain to the hindbrain at intervals of 500 μm, were selected and processed.

### Transmission electron microscopic (TEM)

TEM studies were carried out as described previously [64]. Mice were euthanized and subsequently perfused with 4% glutaraldehyde at 37 °C to fix the tissue. The sensory cortices were then carefully dissected and fixed further in 2% osmium tetroxide in sodium cacodylate buffer. Following fixation, the samples were stained with 2% uranyl acetate, dehydrated in a series of graded ethanol solutions, and finally embedded in epon-araldite resin for ultrathin sectioning. Ultrathin sections were prepared using an Ultramicrotome (Leica Microsystems) and subsequently stained with uranyl acetate and lead citrate for contrast enhancement. For each mouse, six electron microscopy (EM) images were randomly selected for analysis. This analysis was conducted by investigators who were blinded to the experimental subgroups.

### Permeability studies

ECs barrier function was evaluated through the transfer of HRP across EC monolayers. These monolayers were cultured in media supplemented with 10% fetal calf serum (FCS) on polycarbonate filters featuring 3μm pores within a Transwell system, following previously established protocols [65].

### Real-time PCR

The brain cortex was harvested and processed using Trizol for RNA extraction, then real-time PCE was performed following a previously outlined procedure [66]. The *NEO1* mRNA level was normalized to that of *Gapdh*. The primers used were as follows: *NEO1*, CTAGCATTGTAGTGAGCTGGAC (forward) and GCACTGGAGTGTATGGAGCATT (reverse); and *Gapdh*, AGTGCCAGCCTCGTCTCATA (forward) and GATGGTGATGGGTTTCCCGT (reverse).

### Antibodies for western blot and immunofluorescent staining

For western blot analysis, the following primary antibodies were employed: anti-cleaved Caspase3 (1:1000; immunoway), anti-claudin-5 (1:10,000; Proteintech, 66378-1), anti-ZO-1 (1:1000; Cell Signaling Technology, 13663), anti-Neogenin (1:1000; Cell Signaling Technology, 39447), anti-FTH1 (1:1000; Cell Signaling Technology, 4393), anti-FPN1 (1:1000; Proteintech, 26601-1-AP), anti-FTL (1:1000; Proteintech, 10727-1-AP), anti-PECAM-1 (1:500; Servicebio, GB13063-50), anti-hepcidin (1:1000, Abcam, ab190775), anti-β-actin (1:1000; Proteintech, 81115-1-RR). HRP-conjugated secondary antibodies were used.

For immunofluorescent staining, the primary antibodies included: anti-PECAM-1 (1:100; Servicebio, GB13063-50), anti-claudin-5 (1:100; Proteintech, 66378-1), anti-ZO-1 (1:100; Cell Signaling Technology, 13663), anti-GFAP (1:1000; Cell Signaling Technology, 3670), anti-SLC16A1 (1:100; Proteintech, 20139-1-AP), anti-Laminin-γ1 (1:100; DSHB, 2E8), anti-Collagen IV (1:200; abcam, ab6586), anti-Caveolin-1 (1:200; LSBio, Ls-B9776), anti-α-SMA (1:100; Servicebio, GB12045-100). The appropriate secondary antibodies for immunostaining were purchased from Thermo Fisher Scientific. The scanned images were analyzed with ImageJ NIH software.

### Evans Blue extravasation

For the assessment of BBB permeability to Evans Blue, mice were received an intravenous injection of Evans Blue (4 mg/kg) through the caudal vein. After anesthetized with isoflurane and perfusion with heparinized PBS (100 U/mL), the brain cortex was excised and weighed. The brain cortex was then divided: one half was homogenized in PBS with 25% trichloroacetic acid (TCA) for overnight incubation at 4 °C, while the other half was dried at 60 °C for 24 h and subsequently weighed. The homogenate was centrifuged at 1000 × *g* for 30 min at 4 °C. Evans blue standards were prepared in advance and a standard curve was constructed for quantitative analysis. The absorbance of the supernatant was measured at 620 nm using a 96-well plate reader, allowing for the calculation of dye concentration relative to tissue weight.

### Adeno-associated virus (AAV) generation and injection

AAV vector AAV5-GFaABCID-NTN1-myc-6his-GFP was synthesized by BrainVTA. The negative control virus used was AAV5-GFP. The mice were anesthetized with 1% isoflurane and small cranial openings were created on the left side of the skull. The designated injection coordinates were −1.06 and −2.06 mm posterior, 1.5 mm lateral to the bregma, and at a depth of 0.8 mm. One group of mice (n = 4) were injected with 0.2 μl volume of AAV-NTN1, and another group mice (n = 4) were injected with 0.2 μl volume of AAV-GFP using a microliter syringe (Hamilton) at a speed of 200 nl/min controlled by UltraMicroPump. 14 days after injection, the mice were subjected to the SAH model induction. The GFP was stained to verify the successful delivery and expression of the AAV constructs. We utilized dye to validate the injection target in this study.

### Hepcidin injection

For the administration of hepcidin, mice were anesthetized using the previously described method. Based on findings from prior research indicating the superior efficacy of human hepcidin-25 over mouse hepcidin-1 in regulating iron content [67], human hepcidin-25 (Abcam) was used in this experiment. Human hepcidin-25 was formulated with double distilled water to a final concentration of 1 μg/μl. After hemorrhage 1 day, small openings were made in the skull on the hemorrhage side to facilitate injection. A dose of 3 μl of human hepcidin-25 was then directly injected into the cortex at coordinates −1.06 mm and −2.06 mm posterior, 1.5 mm lateral, and 0.8 mm in depth relative to the bregma using a microliter syringe (Hamilton) at a speed of 200 nl/min controlled by UltraMicroPump.

### Mouse brain microvascular endothelial cell (MBMEC) and human umbilical vein endothelial cell (HUVEC) cultures

MBMEC and HUVEC were obtained from Pricella. MBMECs and HUVECs were seeded in a 6-well plate with DMEM supplemented with 10% FBS. For a parallel experiment, the original astrocytes from the control and Neo1 KO mice were cultured up to the 2nd generation, then placed in insert wells containing DMEM with 1% FBS for 24 h. Subsequently, the culture medium harvested from either control or Neo1 KO astrocytes was transferred to the MBMEC or HUVEC 6-well plates (which initially had no culture medium) and maintained for 6 h, with EdU added into the medium (at a final concentration of 3 μg/ml) simultaneously for assessment of cell.

For the EC migration assay, 2 × 10^5^/ml MBMEC or HUVEC were seeded into a 24-well insert (0.4 μm pore size) and cultured for 16 h. The medium within the insert contained 1% FBS DMEM, whereas the medium outside the insert comprised CM from astrocytes (DMEM with 1% FBS previously cultured with control or Neo1 KO astrocytes). To evaluate EC migration, the insert membrane was stained with crystal violet, allowing for the quantification of cells that migrated through the pores by an inverted light microscope (Leica).

### Brain microvascular endothelial cell isolation

The isolation of brain microvascular endothelial cells was performed as previously described [68, 69]. Initially, brains were homogenized in the pre-cooled HBSS buffer solution at 4 °C, maintaining a volume ratio of sample to buffer of 1: 3. The HBSS buffer comprised HEPES 10 mM, NaCl 141 mM, KCl 4 mM, MgSO4•3H2O 1 mM, NaH2PO4•2H2O 1 mM, CaCl2 2.5 mM, glucose 10 mM, and sodium pyruvate 1 mM. The resulting homogenate was combined with an equal volume of 32% dextran solution and centrifuged at 7245× *g* for 15 min at 4 °C. Following centrifugation, the supernatant was discarded, and the pellet containing brain microvasculature was washed with 16% dextran solution in HBSS buffer. After another round of centrifugation, the pellet was resuspended in HBSS buffer and then passed through 100 μm membrane filters to remove larger debris. The final pellet, obtained after another round of centrifugation, was prepared for Western blot analysis. FTH, FTL, and PECAM-1 were to be tested by Western blot.

### RNA-Scope based Situ hybridization

RNA-Scope in situ hybridization was conducted using the HAMP probe, acquired from Ribo, by following the supplied protocol. Original neurons and astrocytes were first seeded into confocal microscopy-compatible culture dishes and allowed to adhere and grow for 24 h. Then, the cells were gently washed three times with PBS and subsequently fixed with 4% PFA for 10 min. Post-fixation, the cells were prepared for hybridization by incubating them with 100 μl of Pre-hybridization Buffer at 37 °C for 30 min. Following the pre-hybridization treatment, the buffer was discarded, and the cells were incubated with 100 μl of the HAMP FISH Probe for specific mRNA detection, or a positive control probe, in a humidified chamber overnight at 37 °C. After washing, the slides were treated with a pre-amplifier for 20 min and an amplifier for 15 min at 40°C. The final step involved imaging the hybridized samples using a confocal microscope at the cy3 channel.

### Statistical analysis

Sample sizes are indicated in the figure legends, and were consistent with previous studies. Statistical analysis was conducted using GraphPad Prism 9, with investigators blinded to subgroup allocation. Data are expressed as mean ± SD. Pearson analysis was used for correlation assessments. Differences between the two groups were analyzed using the Student’s *t*-test and Mann-Whitney *U* test. The normally distributed data were analyzed by one-way ANOVA followed by Bonferroni’s or Fisher’s least significant difference post hoc tests. The exact *P* value for each analysis is shown in the corresponding figure or figure legend. *P* < 0.05 was considered significant.

### Supplementary information

Supplementary Materials

Original data

## Data Availability

All data needed to evaluate the conclusion in the paper are present in the paper and/or the Supplementary Materials. Full and uncropped western blots are present in the Supplementary Materials.
