# Supplementary material for: Hepcidin depending on astrocytic NEO1 ameliorates blood-brain barrier dysfunction after subarachnoid hemorrhage
Source: Cell Death Dis. 2024 Aug 7;15(8):569. doi: 10.1038/s41419-024-06909-x (PMC11303805; doi:10.1038/s41419-024-06909-x)
Supplement: Supplementary file 1 — Supplementary Materials [file 41419_2024_6909_MOESM1_ESM.docx]

**Supplementary Text**

**Methods**

**Analysis of CSF mass spectrometry**

**1. Construction of protein regulatory network**

In order to construct a regulatory network between differentially expressed proteins and target genes, human protein protein interaction data was obtained from public web server CMGRN ^1^ and PTHGRN ^2^. Dijkstra algorithm is used to calculate the shortest propagation path of differentially expressed proteins to target genes in human protein interaction networks.

$$Algorithm:Dijkstra$$

$$Input:Directed graph G=\left( V, E,W \right) with weight$$

$$Output:All the shortest paths from the source vertex s to every vertex v_{i}\in V\backslash\left\{ s \right\}$$

$$1:S\leftarrow\left\{ s \right\}$$

$$2:dist\left[ s,s \right]\leftarrow0$$

$$3:\boldsymbol{for} v_{i}\in V-\left\{ s \right\} \boldsymbol{do}$$

$$4: dist\left[ s,v_{i} \right]\leftarrow w\left( s,v_{i} \right)$$

$(when v_{i} not found, dist\left[ s,v_{i} \right]\leftarrow\infty$)

$$5:\boldsymbol{while} V-S\neq\emptyset\boldsymbol{do}$$

$$6: find \min_{v_{i}\in V} dist\left[ s,v_{i} \right] from the set V-S$$

$$7: S\leftarrow S\cup\left\{ v_{j} \right\}$$

$$8: \boldsymbol{for} v_{i}\in V-S \boldsymbol{do}$$

$$9: \boldsymbol{if} dist\left[ s,v_{i} \right]+w_{i,j}<dist\left[ s,v_{i} \right] \boldsymbol{then}$$

$$10: dist\left[ s,v_{i} \right]\leftarrow dist\left[ s,v_{i} \right]+w_{i,j}$$

$G=\left( V, E,W \right)$ represents a weighted undirected graph. Where the weight of each edge $e_{i,j}:=\left\{ v_{i},v_{j} \right\}$is a non-negative real number $w_{i,j}\left( e_{i,j} \right)$, which represents the distance from the vertex $v_{i}$ to the vertex $v_{j}$. And set a single source point $s\in V$. Find the shortest path from the origin s to all nodes in $V\backslash\left\{ s \right\}$.

1. At the beginning of the algorithm, the selected source point $s$ is put into the set $S$;

2. The shortest path from the source point $s$ without a self-loop to itself is 0;

3. When the vertex $v_{i}$ is not in the set $S$ (at this time, there is still only the source point $s$ in the set $S$), it begins to enter the loop;

4. Assign the weight between the source point $s$ and the point $v_{i}$ to $dist\left[ s,v_{i} \right]$. Because it is a directed graph, $dist\left[ s,v_{i} \right]\leftarrow\infty$ when the source point $s$ does not point to the vertex outside any other set $S$. It can be understood that starting from the source point $s$ at this time, $v_{i}$ cannot be reached temporarily. However, with the expansion of the set $S$, all the vertices can be reached from the source point $s$. At this point, the first $\boldsymbol{for}$ loop ends.

5. If the set $V-S$ is not an empty set, enter a loop;

6. Select the vertex $v_{j}$ which is in the set $V-S$ after the first $\boldsymbol{for}$ cycle and has the shortest distance relative to the set $S$;

7. merge this vertex $v_{j}$ into the set $S$, so as to achieve the purpose of expanding the set $S$;

8. After merging vertex $v_{i}$ into set $S$, it may affect the length of the shortest path of other vertices relative to set $S$, so entering the inner $\boldsymbol{for}$ loop will update the influential ones;

9. That is, if the length of the shortest path from the source point $s$ to the vertex $v_{j}$ we selected in step 6 plus the distance $w_{i,j}$ from the vertex $v_{j}$ to the vertex $v_{i}$ is smaller than the length of the shortest path from the source point $s$ to the vertex $v_{i}$ relative to the set $S$;

10. Update the shortest path from the source point $s$ to the vertex $v_{i}$ relative to the set $S$ to the shortest path from the source point $s$ to the vertex $v_{j}$ selected by us in step 6 plus the weight $w_{i,j}$ between the vertex $v_{j}$ and the vertex $v_{i}$.

**2. Protein correlation calculation**

Use R language 3.6.0 to calculate Pearson correlation coefficient between protein.

**3. Visualization of protein regulatory network**

Visualization of differentially expressed protein-target gene regulatory network by Cytoscape 3.9.0.

**Western blotting**

Western blotting was performed as our previous study^3^. The parietal cortex samples from the bleeding side were rapidly collected after perfusion with cooled 0.9% saline. RIPA lysis buffer (Beyotime) was used to abstract the proteins. The lysates were sonicated and centrifuged for 15 minutes at 4℃ and the supernatant was obtained. A bicinchoninic acid protein assay kit (Solarbio) was used to detect the total protein concentration. Equal amounts of protein samples (40 µg) were used for sodium dodecyl sulfate-polyacrylamide gel electrophoresis and the proteins were then transferred to a polyvinylidene difluoride filter membrane (Millipore Sigma, ISEQ00010). The membranes were then immersed with 5% non-fat milk for 1 hour at ambient temperature and subsequently incubated at 4°C overnight with the relevant antibodies. The intensities of blots were analyzed with ImageJ software 1.5 (National Institutes of Health, Bethesda, MD, USA).

**IF staining**For immunostaining analysis, brain sections were incubated with a blocking solution
containing 0.1% Triton X-100 for permeabilization and 10% donkey serum for 20 minutes at room temperature and then with primary antibody (in PBS with 10% donkey serum) at 4°C overnight. The following primary antibodies were used in Antibodies for western blot and immunofluorescent staining. The brain
sections were washed with PBST buffer, and incubated with appropriate
secondary antibodies at 1:500 (Thermo Fisher Scientific, Alexa Fluor conjugates) for
2h at room temperature. Brain sections were washed and mounted using Antifade Mounting Medium (Beyotime). The images were taken by a Nikon confocal microscope with 10×, 20×, or 40× objective, with a sequential-acquisition setting. Three slices were randomly selected from each brain, and then three random fields in the parietal cortex of each slice were selected. Quantitative analyses were performed using ImageJ software and ImageJ-vessel analysis. To precisely analyze vessel lengths, Neuron J software was used to trace each single vessel.


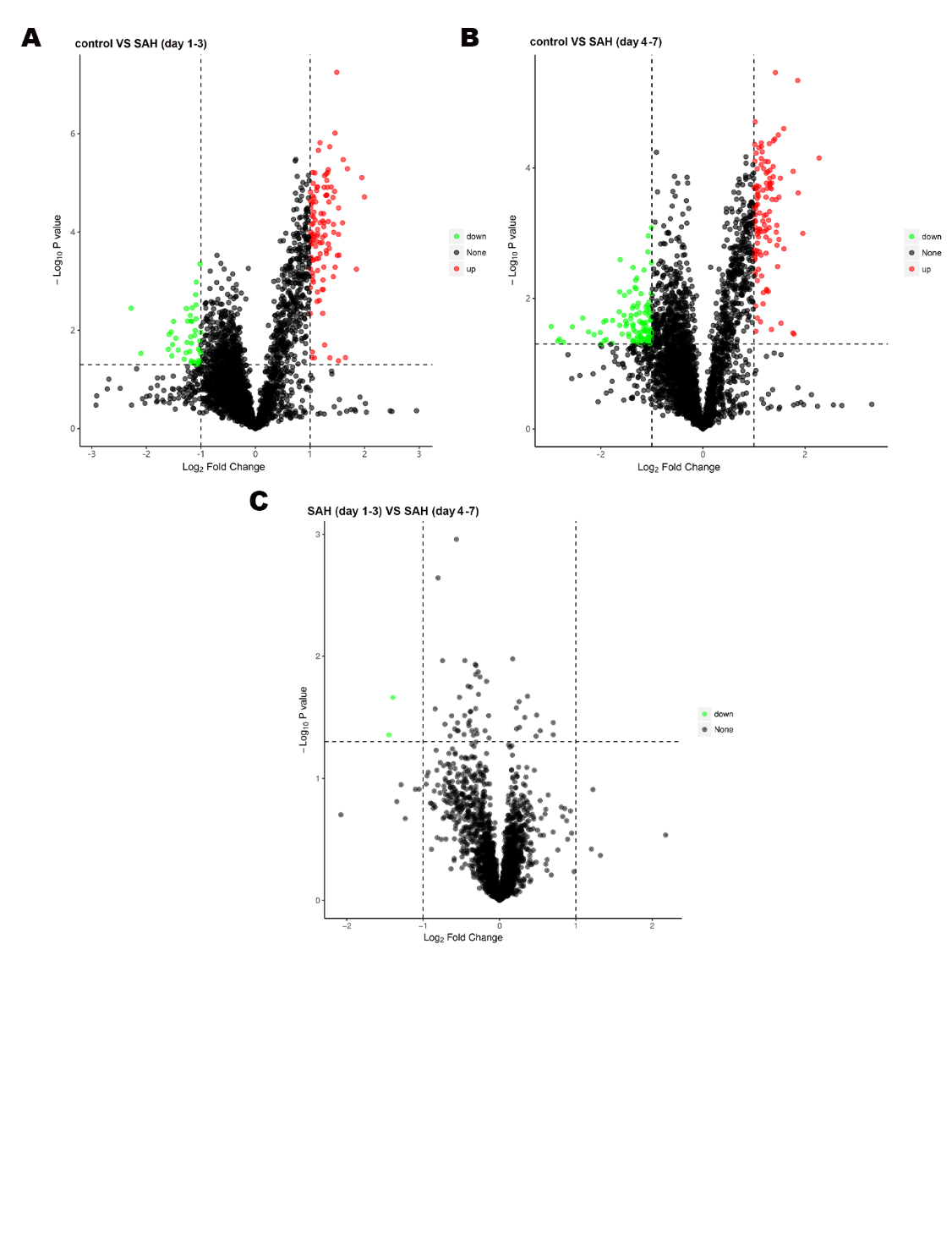


**Fig.S1. The volcano map of different expression proteins in the multiple groups.** (A) The volcano map of different expression proteins between control and SAH (days 1-3). (B) The volcano map of different expression proteins between control and SAH (days 4-7). (C) The volcano map of different expression proteins between SAH (days 1-3) and SAH (days 4-7).


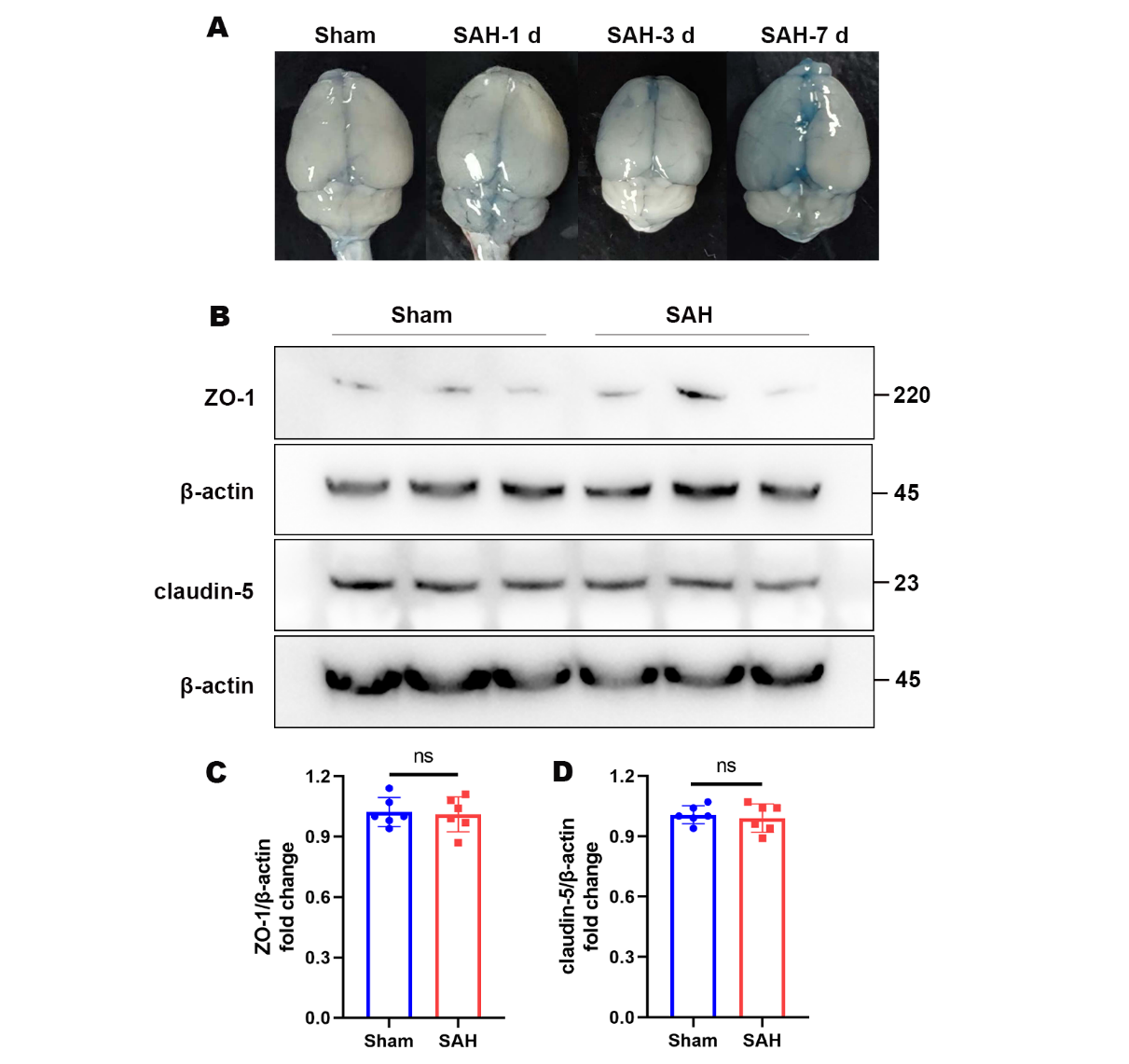


**Fig.S2. The BBB disruption and ECs junction in SAH cortex. (A) Investigate the BBB disruption in the cortex by using EB.** The EB showed blue. (B) Representative immunoblotting of ZO-1 and claudin-5 in the cortex of the sham and SAH-7 days. (C and D) Quantification analysis of data in B *(n* = 6 mice/group). β-actin was used as a loading control. Data are presented as mean ± SD. ns, not significant. Student’s *t* test.


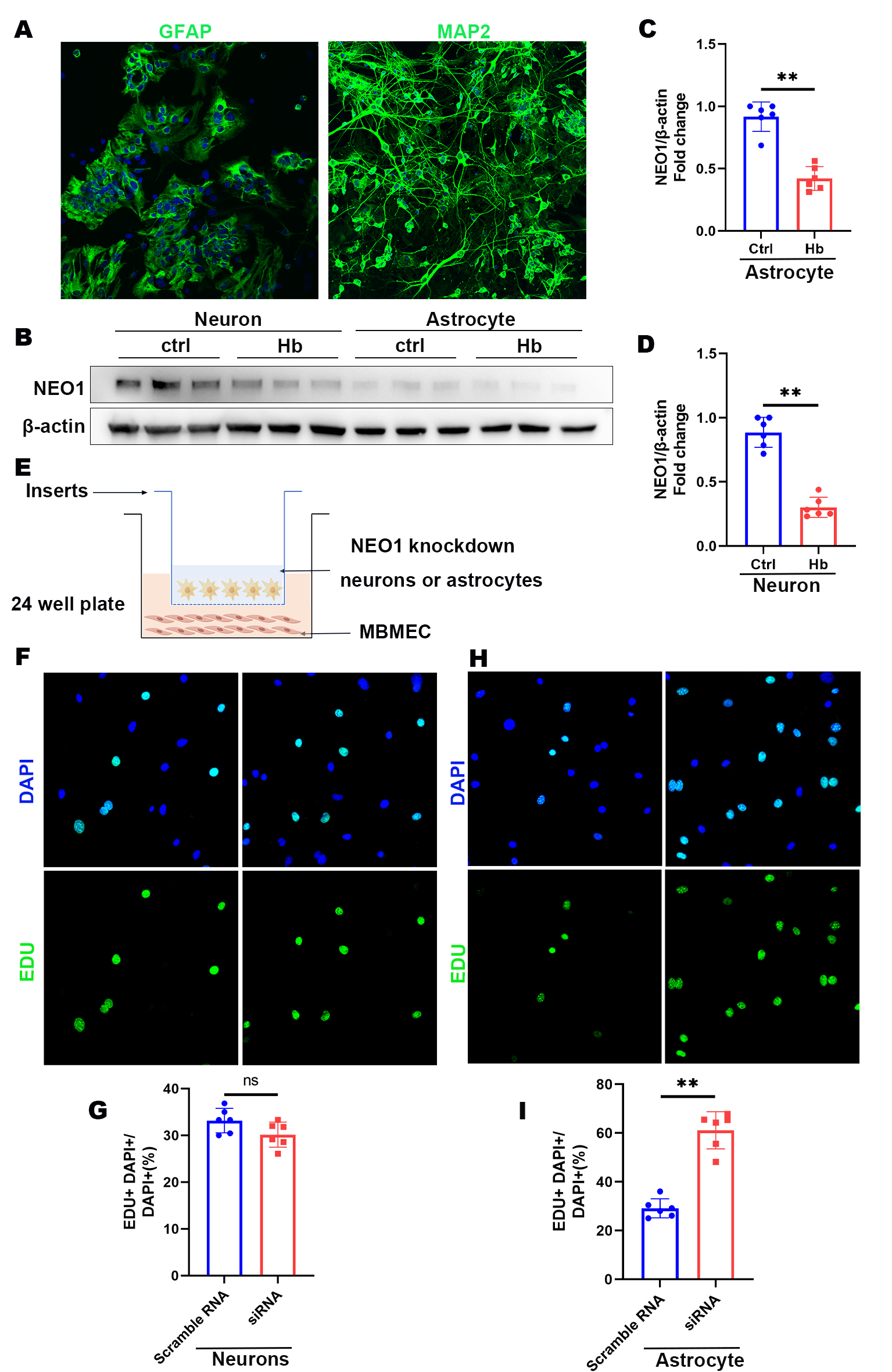


**Fig.S3. Increased EC proliferation in MBMEC cultures exposed to NEO1 knockdown astrocytes and detected expression of NEO1 in Hb-treated astrocytes and neurons.** (A) Representative identification picture of primary astrocytes and primary neurons. (B) Representative immunoblotting of NEO1 and quantification (C and D) of Hb on NEO1 in primary astrocytes and primary neurons (*n* = 6 experiments/group). β-actin was used as a loading control. (E-I) Increased EC proliferation in MBMEC cocultured with NEO1 knockdown astrocytes. (E) Schematic of coculture of MBMEC with NEO1 knockdown neurons or NEO1 knockdown astrocytes. EDU incubated for 4 hours. (F and H) Representative images of EDU+ MBMEC. (G) Quantitative analysis of data in E (*n* = 6 experiments/group). (I) Quantitative analysis of data in G. Data are presented as mean ± SD. ***P* < 0.01; ns, not significant. Student’s *t* test.


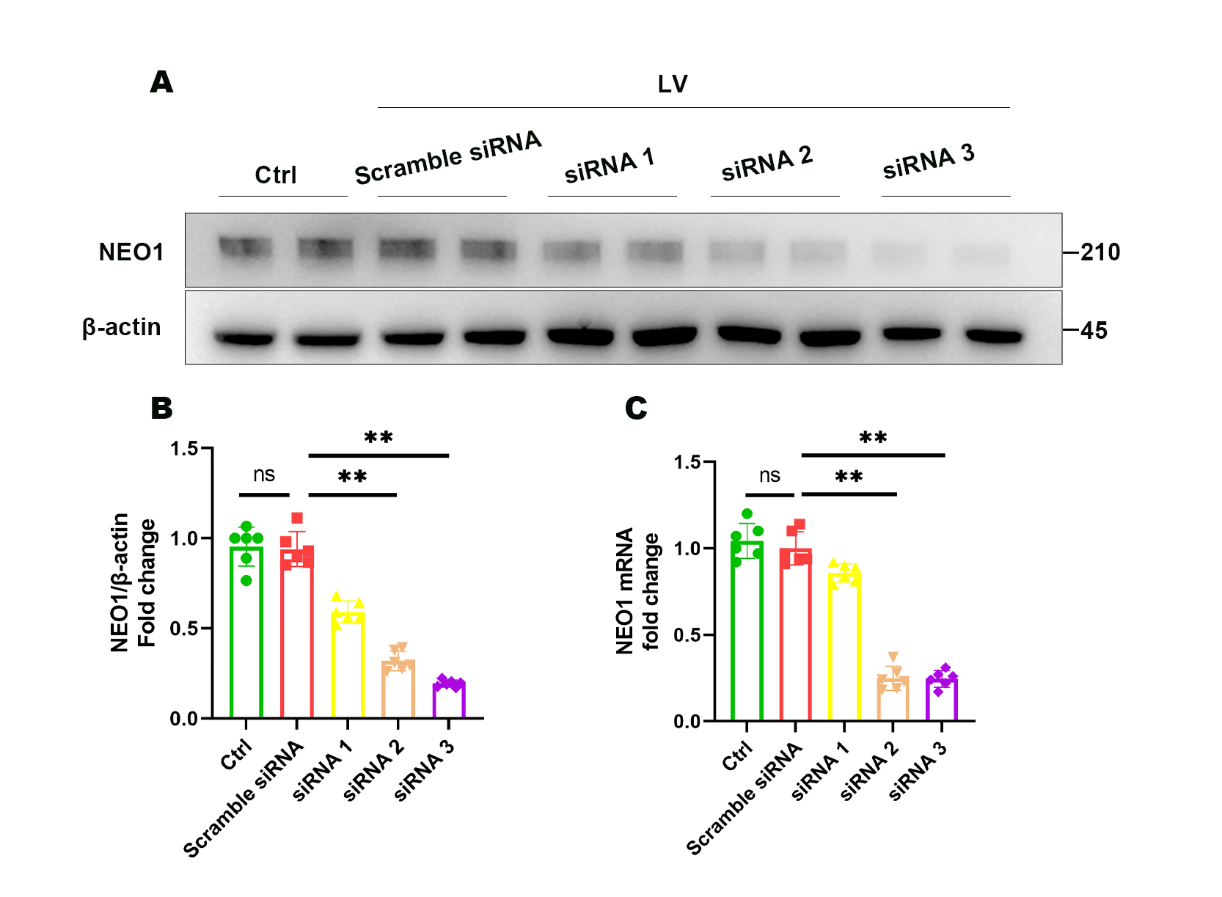


**Fig.S4. Efficiency of LV-siRNA.** (A) Representative immunoblotting of NEO1 and quantification (B) of LV-siRNA on NEO1 (*n* = 6 experiments/group). β-actin was used as a loading control. (C) Total RNA was extracted and the expression of NEO1 was quantitated by RT-qPCR (*n* = 6 experiments/group). The expression level of NEO1 was normalized against β-actin. Data are presented as mean ± SD. **P* < 0.05; ***P* < 0.01. 1-way ANOVA with Tukey’s correction for multiple comparisons.


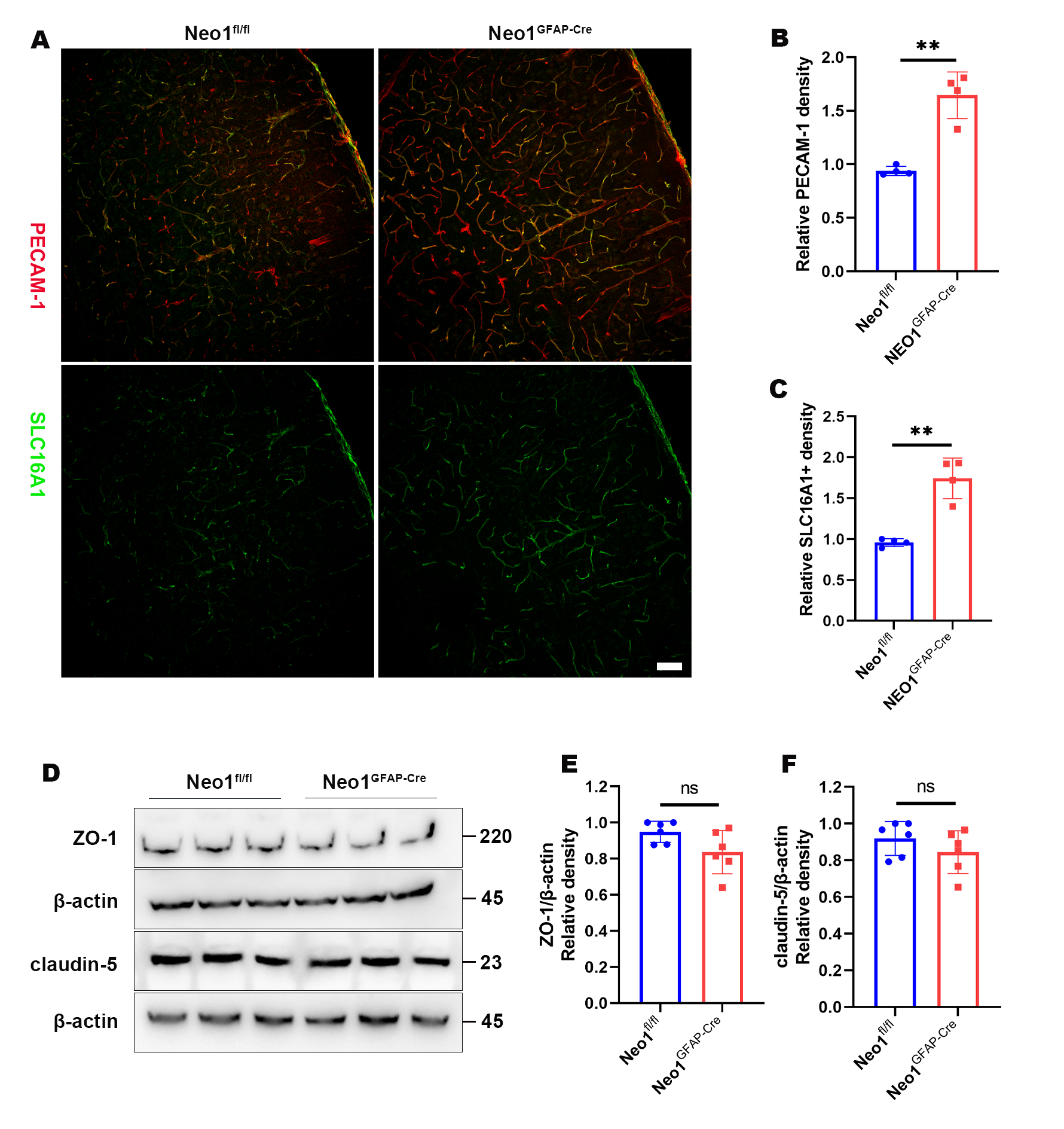


**Fig.S5. Increased vein capillaries, few changes in EC tight junctions in NEO1^GFAP-Cre^ cortex.** (A) Representative images of coimmunostaining analyses using PECAM-1 and SLC16A1 antibodies in NEO1^fl/fl^ and NEO1^GFAP-Cre^ cortex. (B and C) Quantitative analysis data in A. (D) Representative immunoblotting of ZO-1 and claudin-5. (E and F) Quantitative analysis of data in D. Data are presented as mean ± SD. ***P* < 0.01; ns, not significant. Student’s *t* test.


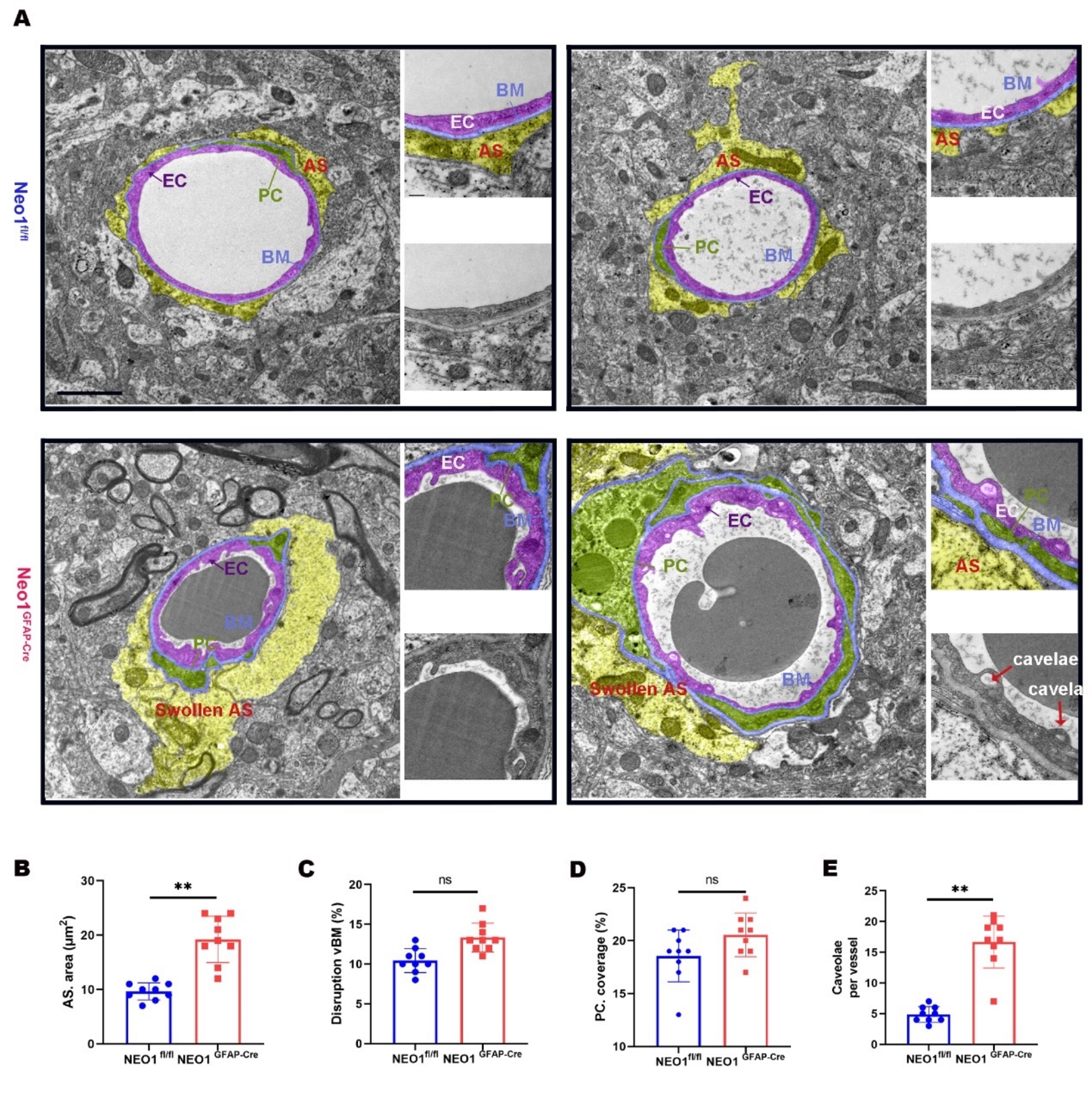


**Fig.S6. Swollen astrocytes, and increased EC caveolae in NEO1 GFAP-Cre cortex by EM analysis.** (A) Representative EM images of blood vessels in NEO1^fl/fl^ and NEO1^GFAP-Cre^ cortex. Astrocytes are highlighted in yellow, pericytes (PC) in green, vBM in blue, and EC in purple. Scale bars: 1 μm. (B) Quantification of astrocytes area. (C) Quantification of disrupted vBMs. (D) Quantification of PC coverage. (E) Quantification of EC caveolae per vessel. Data are presented as mean ± SD (*n* = 3 vessels from 3 mice per group). ***P* < 0.01; ns, not significant. Student’s *t* test.


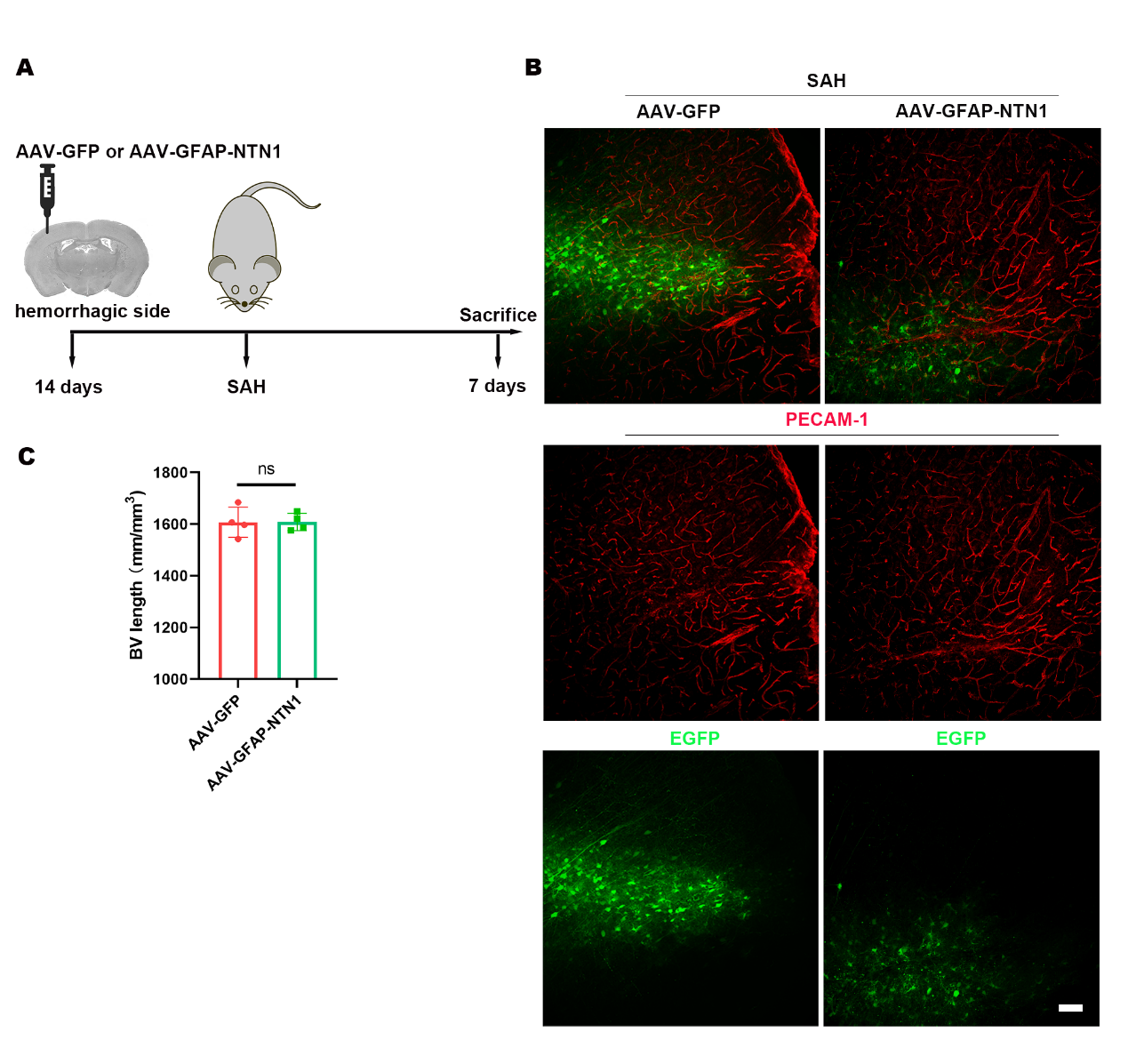


**Fig.S7. NTN1 can’t inhibit the BV density increase in the SAH model cortex.** (A) Schematic of AAV-GFP or AAV-GFAP-NTN1 administration in SAH cortex. AAV-GFP or AAV-GFAP-NTN1 was injected into the hemorrhagic side cortex of the SAH mice model. (B) Representative images of immunostaining PECAM-1 in the cortex. (C) Quantitative analysis of BV length (*n* = 4 mice/group). Data are presented as mean ± SD. ***P* < 0.01; ns, not significant. Student’s *t* test.

**
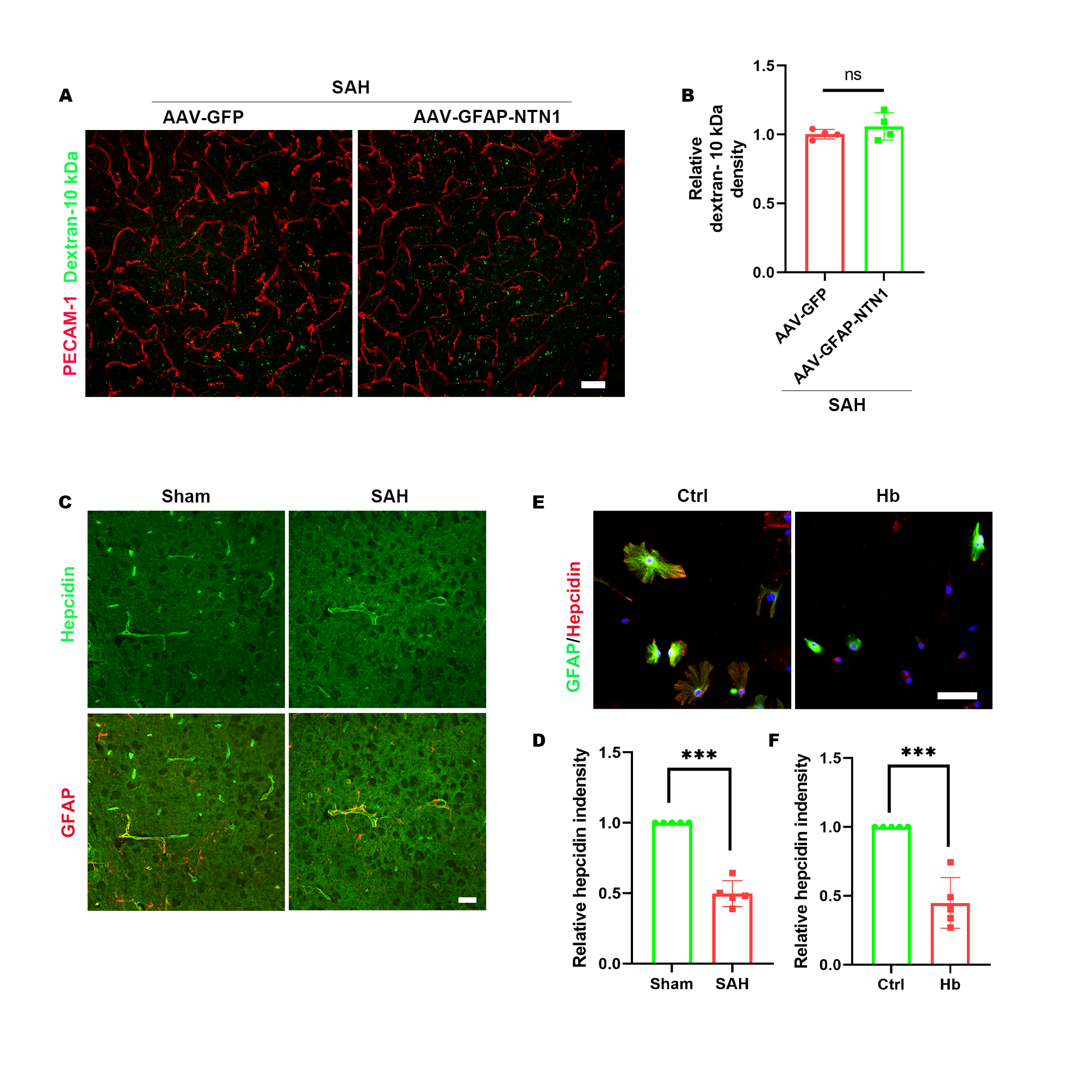
Fig.S8. NTN1 can’t inhibit the BBB leakage in the SAH model cortex and hepcidin decreased in SAH model cortex.** (A) Representative images of dextran 10kDa leakage. (B) Quantitative analysis of data in A (*n* = 4 mice/group). (C) Representative images of hepcidin and astrocytes. (D) Quantitative analysis of data in C. (E) Representative images of hepcidin in original astrocyte vitro model. (F) Quantitative analysis of data in E. Data are presented as mean ± SD. ns, not significant. Student’s *t* test.


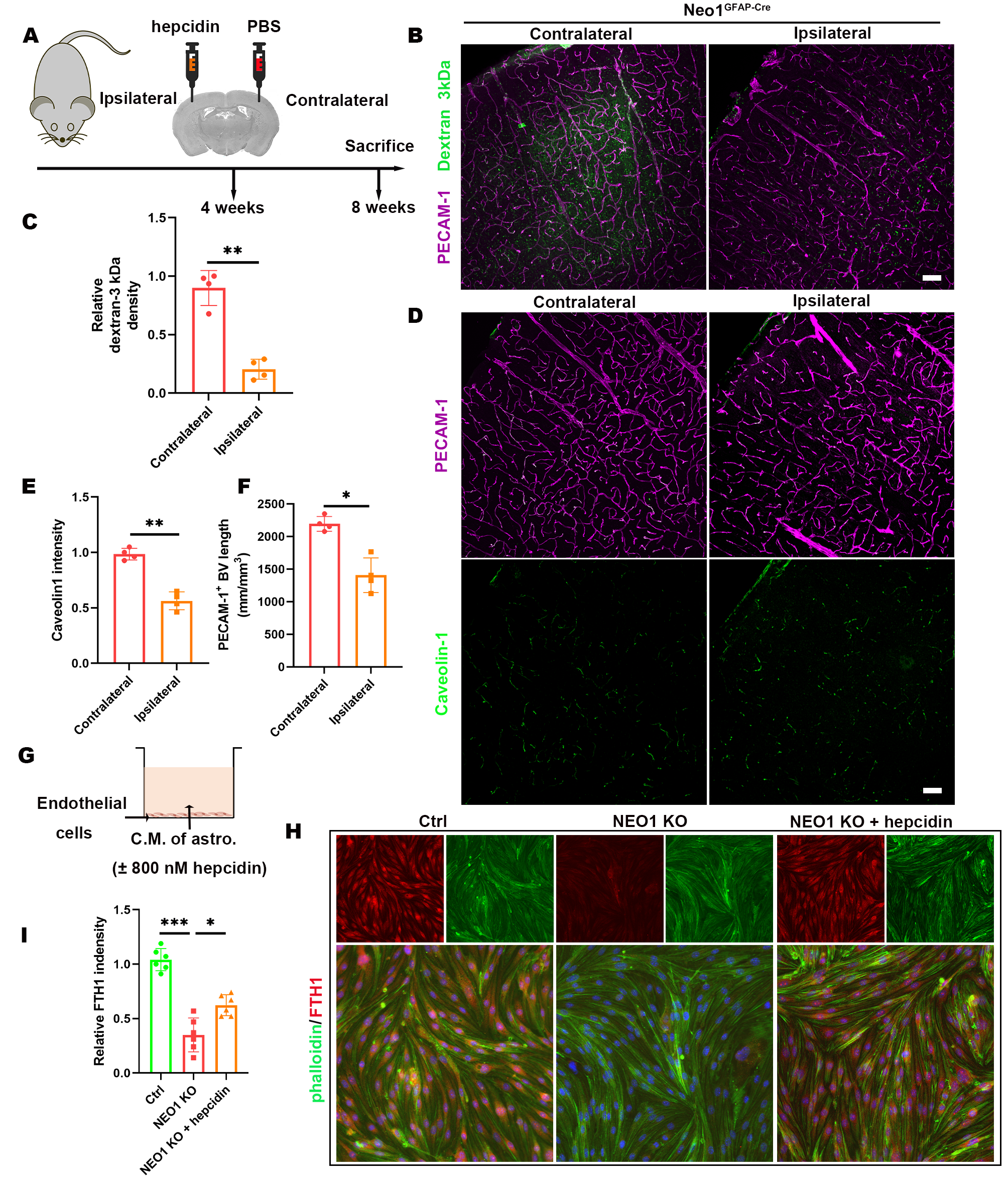


**Fig.S9. Hepcidin amelioration of blood vessel increase and BBB leakage in NEO1^GFAP-Cre^ cortex.** (A) Schematic of hepcidin administration in NEO1^GFAP-Cre^ cortex. Hepcidin was injected into the left side (ipsilateral) of NEO1^GFAP-Cre^. (B) Representative images of dextran 3kDa leakage in ipsilateral and contralateral cortices. (C) Quantitative analysis of data in B. Data are presented as mean ± SD (*n* = 4 mice/group). (D) Representative images showing caveolin-1 staining in the ipsilateral and contralateral cortices. (E and F) Quantitative analyses of caveolin-1 and BV length. (G-I) Hepcidin increase iron content of MBMEC. (G) Schematic of hepcidin administration in MBMEC cultures in the presence of CM of NEO1 KO astrocytes. (H) Representative images of FTH1 of MBMEC. (I) Quantitative analysis of data in H. Data are presented as mean ± SD (*n* = 6 experiments/group). Scale bars: 20 μm. **P* < 0.05; ***P* < 0.01. Student’s t test.

Table S1：Patients’ characteristics

| Baseline data | n=111 |
| --- | --- |
| Sex (Male/Female) | 53/58 |
| Diabetes, n (%) | 0.07 |
| Hypertension, n (%) | 0.34 |
| Clinical data |  |
| High Hunt-Hess (3-4), n (%) | 0.34 |
| High Fisher Grade (3-4), n (%) | 0.61 |
| Had stroke before (Y/N) | 5/106 |
| Outcome |  |
| Length of hospital stay | 18.57±15.69 |

1. Guan D, Shao J, Deng Y, Wang P, Zhao Z, Liang Y*, et al.* CMGRN: a web server for constructing multilevel gene regulatory networks using ChIP-seq and gene expression data. *Bioinformatics* 2014, **30**(8)**:** 1190-1192.

2. Guan D, Shao J, Zhao Z, Wang P, Qin J, Deng Y*, et al.* PTHGRN: unraveling post-translational hierarchical gene regulatory networks using PPI, ChIP-seq and gene expression data. *Nucleic Acids Res* 2014, **42**(Web Server issue)**:** W130-136.

3. Wei B, Liu W, Jin L, Guo S, Fan H, Jin F*, et al.* Dexmedetomidine Inhibits Gasdermin D-Induced Pyroptosis via the PI3K/AKT/GSK3β Pathway to Attenuate Neuroinflammation in Early Brain Injury After Subarachnoid Hemorrhage in Rats. *Frontiers In Cellular Neuroscience* 2022, **16:** 899484.
