# Supplementary material for: Hepcidin depending on astrocytic NEO1 ameliorates blood-brain barrier dysfunction after subarachnoid hemorrhage
Source: Cell Death Dis. 2024 Aug 7;15(8):569. doi: 10.1038/s41419-024-06909-x (PMC11303805; doi:10.1038/s41419-024-06909-x)

Figure 4B unedited gel

β-actin

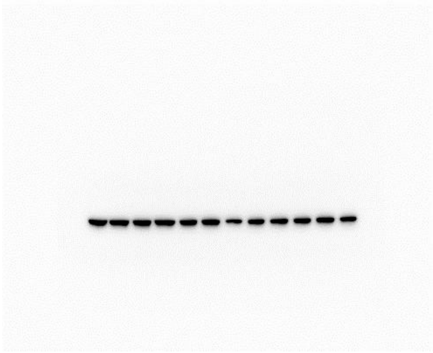

NEO1

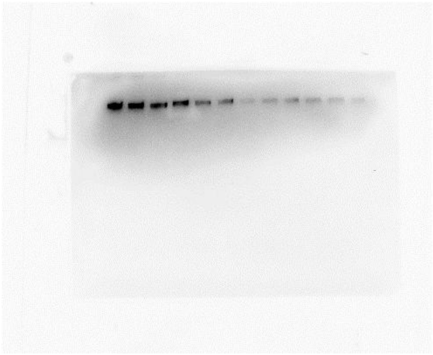

Figure 4N unedited gel

Cleaved-caspase3

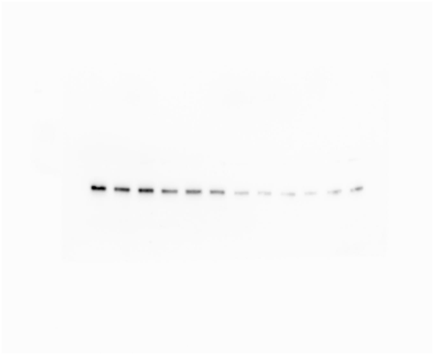

β-actin

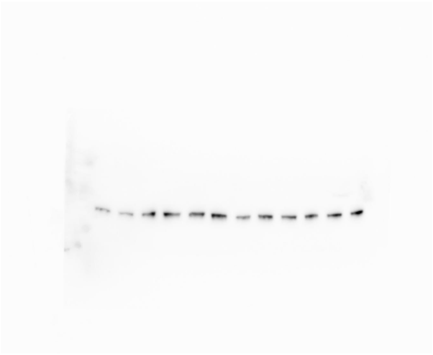

Figure 6K unedited gel

FTH

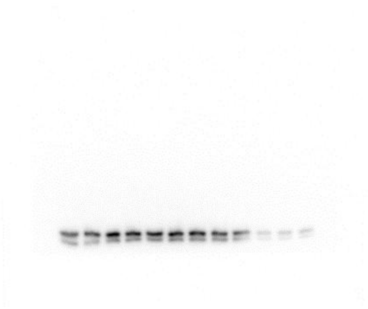

PECAM-1

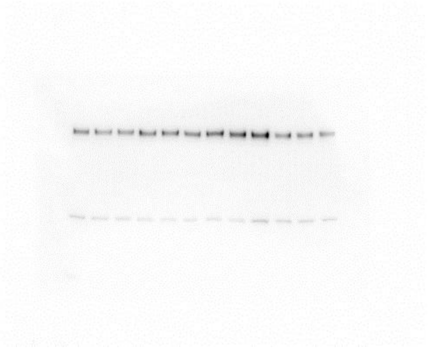

FTL

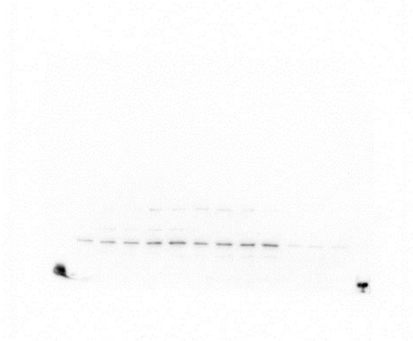

Figure 6N unedited gel

PECAM-1

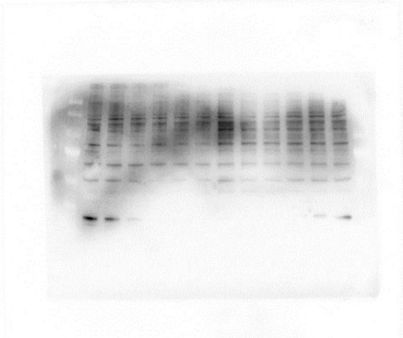

FPN1

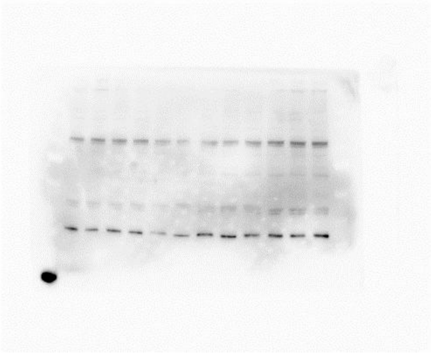

Figure 6P unedited gel

Hepcidin

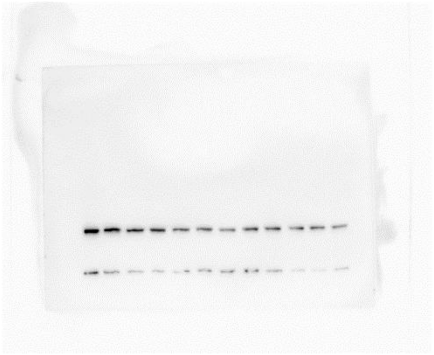

β-actin

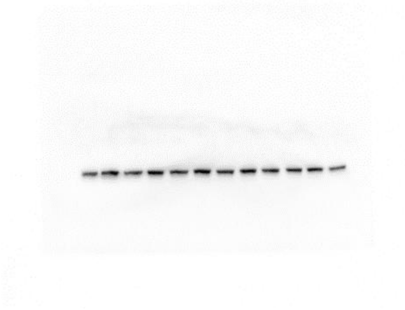

Figure 6V unedited gel

Hepcidin

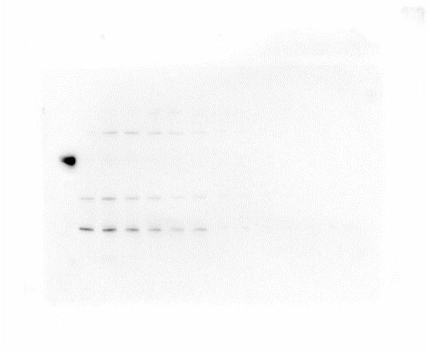

β-actin

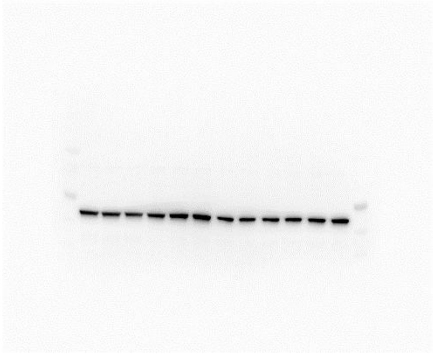

Figure 7A unedited gel

FTH

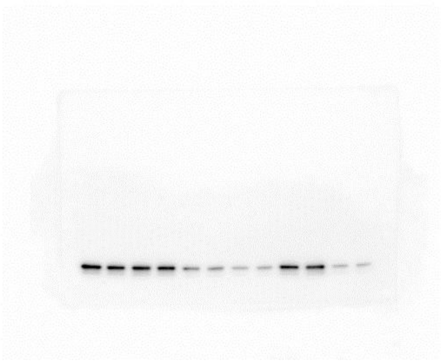

FTL

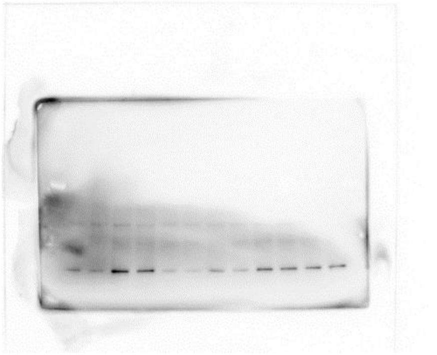

β-actin

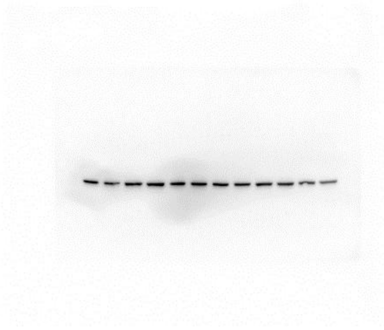

Figure 8B unedited gel

PECAM-1

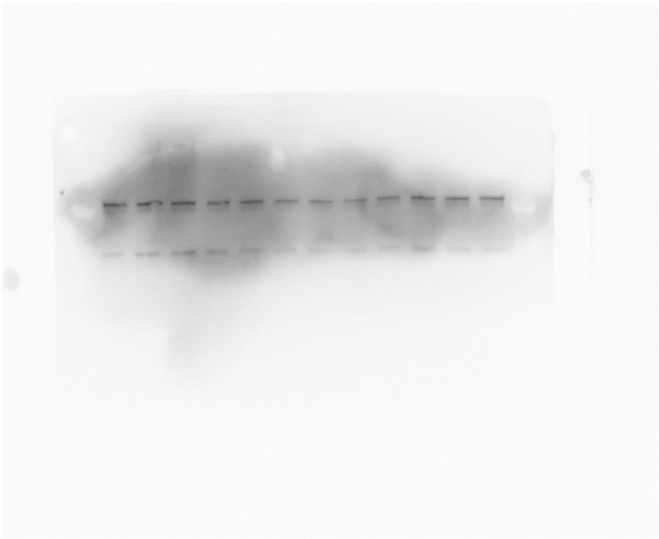

FTH

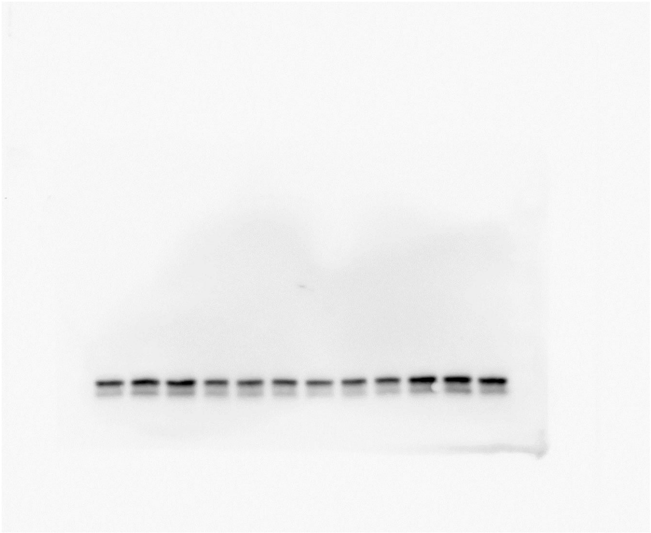

FTL

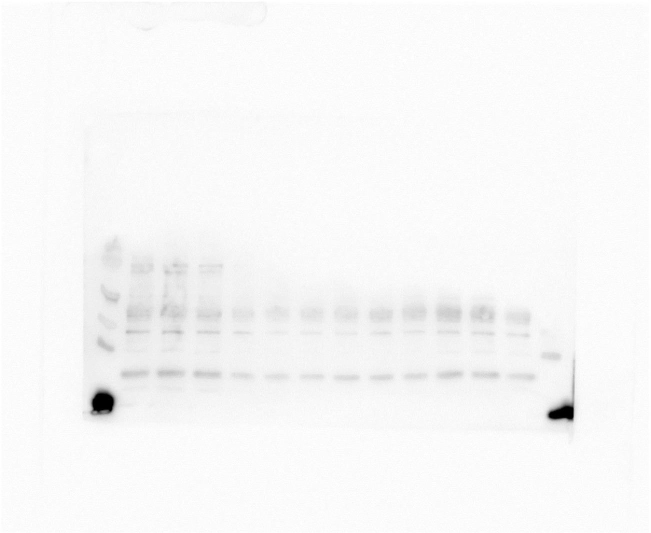

Figure 8E unedited gel

PECAM-1

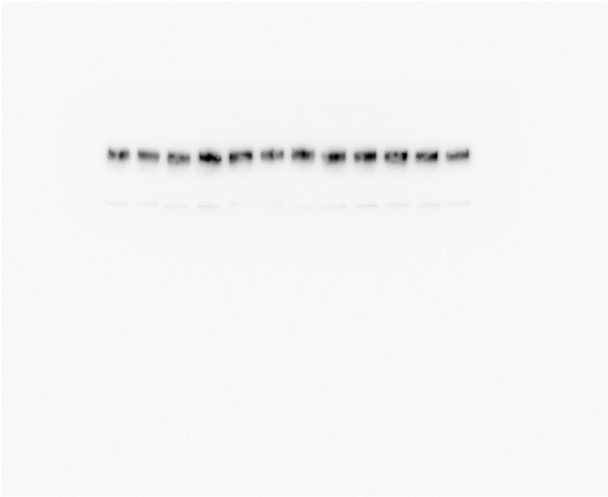

FPN1

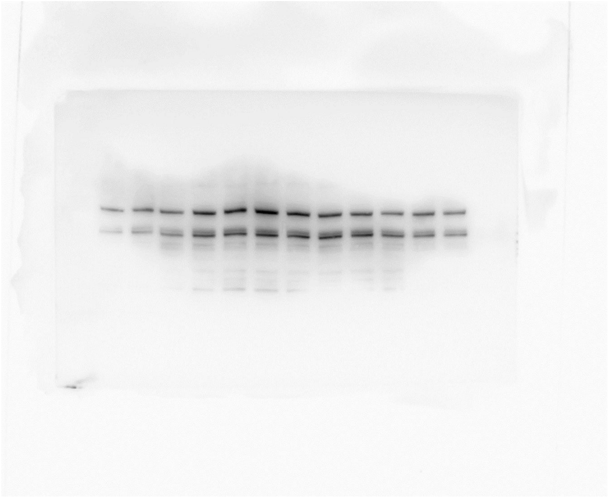

β-actin

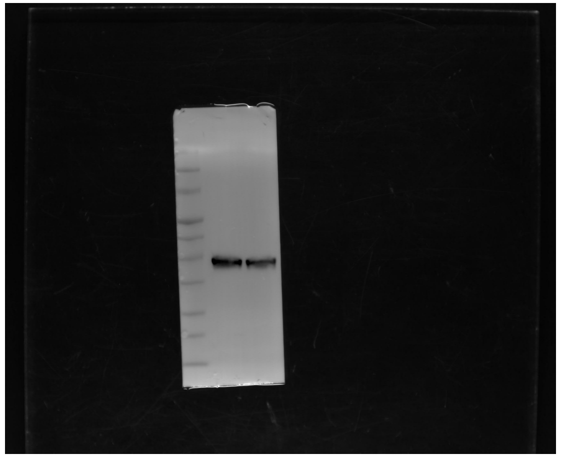

FPN

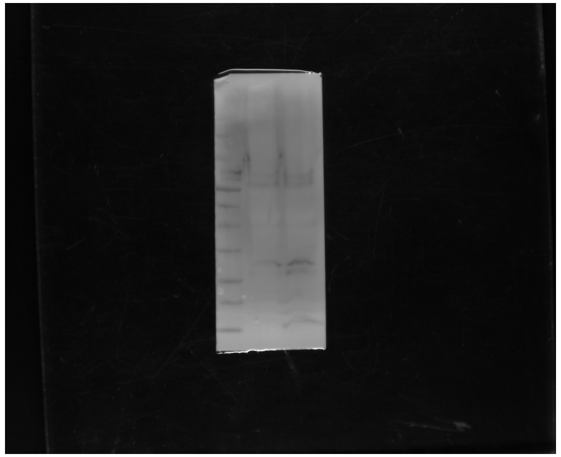

FTH

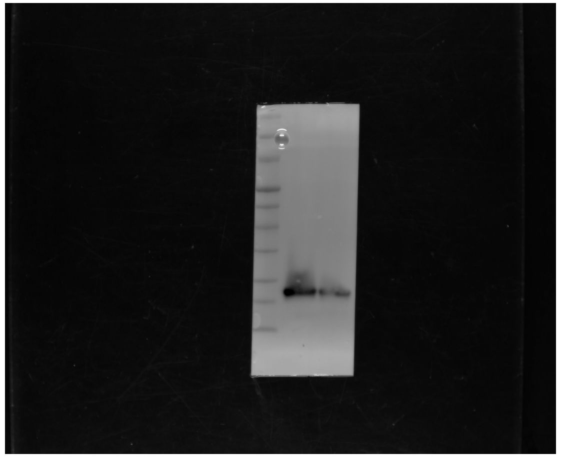

FTL

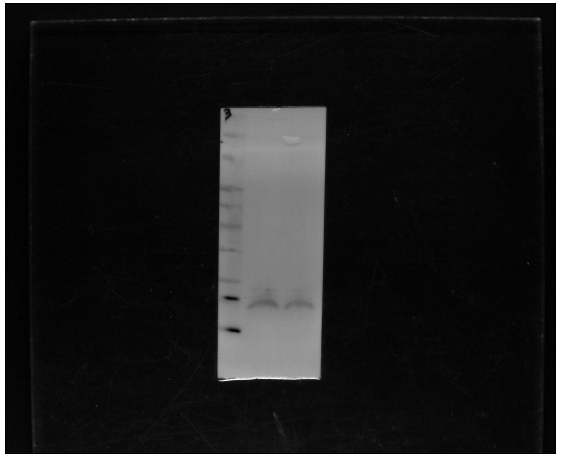

Hepcidin

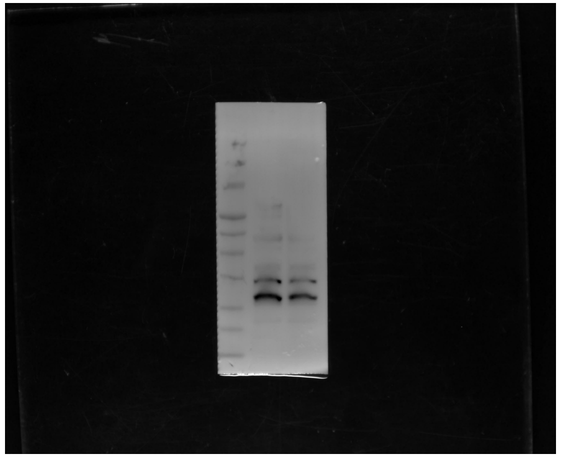

NEO1

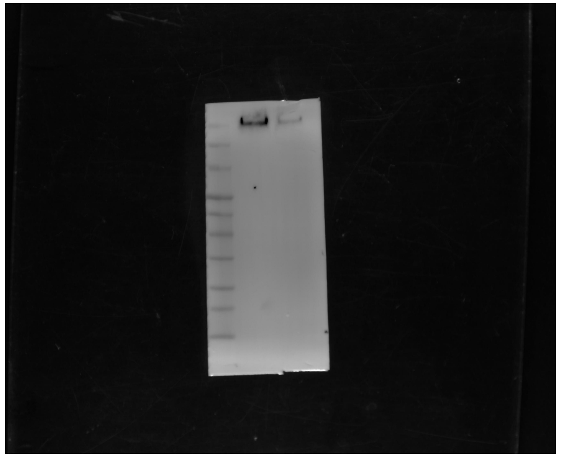

ZO-1

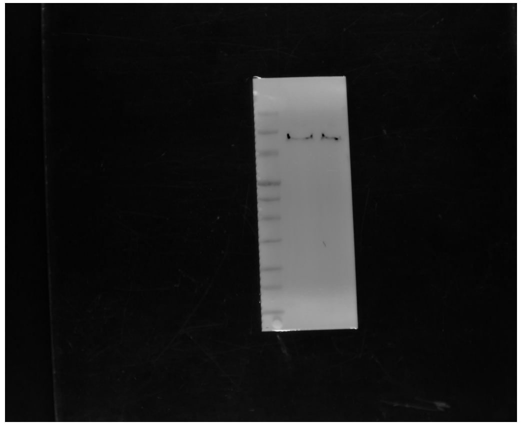

PECAM-1

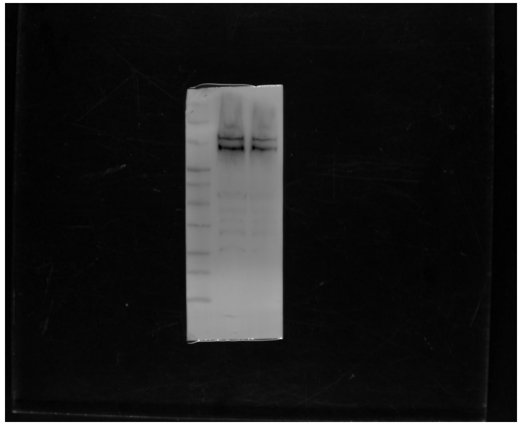

Cleaved-
caspase-3

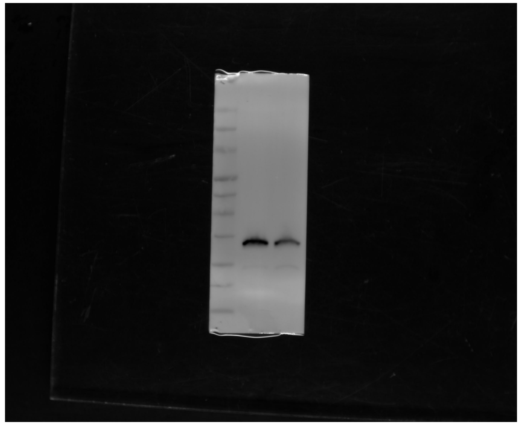

Claudin-5

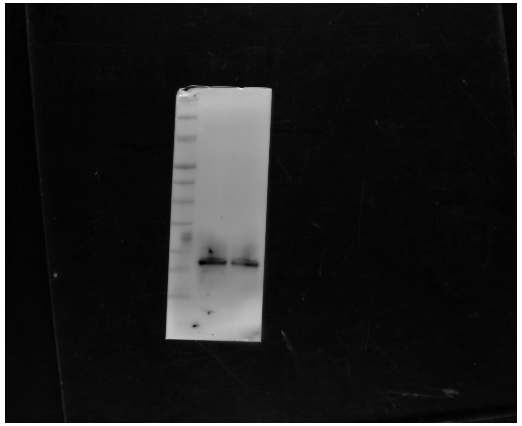

Supplement: Supplementary file 2 — Original data [file 41419_2024_6909_MOESM2_ESM.pdf]
